# HDAC6 inhibition restores TDP‐43 pathology and axonal transport defects in human motor neurons with *TARDBP* mutations

**DOI:** 10.15252/embj.2020106177

**Published:** 2021-03-10

**Authors:** Raheem Fazal, Steven Boeynaems, Ann Swijsen, Mathias De Decker, Laura Fumagalli, Matthieu Moisse, Joni Vanneste, Wenting Guo, Ruben Boon, Thomas Vercruysse, Kristel Eggermont, Bart Swinnen, Jimmy Beckers, Donya Pakravan, Tijs Vandoorne, Pieter Vanden Berghe, Catherine Verfaillie, Ludo Van Den Bosch, Philip Van Damme

**Affiliations:** ^1^ Department of Neurosciences, Experimental Neurology Leuven Brain Institute (LBI) KU Leuven – University of Leuven Leuven Belgium; ^2^ Center for Brain & Disease Research Laboratory of Neurobiology VIB Leuven Belgium; ^3^ Department of Genetics Stanford University School of Medicine Stanford CA USA; ^4^ Stem Cell Institute Department of Development and Regeneration Stem Cell Biology and Embryology KU Leuven Leuven Belgium; ^5^ Department of Microbiology, Immunology and Transplantation Laboratory of Virology and Chemotherapy Rega Institute for Medical Research KU Leuven Leuven Belgium; ^6^ Department of Neurology University Hospitals Leuven Leuven Belgium; ^7^ Department of Chronic Diseases, Metabolism and Ageing Translational Research in GastroIntestinal Disorders, KU Leuven Leuven Belgium

**Keywords:** axonal transport, HDAC6, induced pluripotent stem cells, TDP‐43‐ALS, wild‐type‐ and mutant‐tagged TDP‐43, Molecular Biology of Disease, Neuroscience

## Abstract

TDP‐43 is the major component of pathological inclusions in most ALS patients and in up to 50% of patients with frontotemporal dementia (FTD). Heterozygous missense mutations in *TARDBP*, the gene encoding TDP‐43, are one of the common causes of familial ALS. In this study, we investigate TDP‐43 protein behavior in induced pluripotent stem cell (iPSC)‐derived motor neurons from three ALS patients with different *TARDBP* mutations, three healthy controls and an isogenic control. *TARDPB* mutations induce several TDP‐43 changes in spinal motor neurons, including cytoplasmic mislocalization and accumulation of insoluble TDP‐43, C‐terminal fragments, and phospho‐TDP‐43. By generating iPSC lines with allele‐specific tagging of TDP‐43, we find that mutant TDP‐43 initiates the observed disease phenotypes and has an altered interactome as indicated by mass spectrometry. Our findings also indicate that TDP‐43 proteinopathy results in a defect in mitochondrial transport. Lastly, we show that pharmacological inhibition of histone deacetylase 6 (HDAC6) restores the observed TDP‐43 pathologies and the axonal mitochondrial motility, suggesting that HDAC6 inhibition may be an interesting therapeutic target for neurodegenerative disorders linked to TDP‐43 pathology.

## Graphical abstract

Pathological hallmarks and axonal transport defects observed in mutant TDP‐43 iPSC‐derived motor neurons can be rescued by genetic correction of the mutation using CRISPR/Cas9 or by treatment with a selective HDAC6 inhibitor.

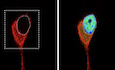

## Introduction

Amyotrophic lateral sclerosis (ALS) is an adult‐onset neurodegenerative disorder with a lifetime risk of 1 in 300 (Brown & Al‐Chalabi, 2017; van Es, 2017). Motor neurons in the motor cortex, brainstem and spinal cord degenerate, causing progressive limb muscle weakness, dysarthria, dysphagia, and respiratory insufficiency. Patients generally die within 2–5 years after symptom onset, mainly due to respiratory and bulbar dysfunction. Riluzole and edaravone are the only FDA‐approved drugs, yet with limited effect on the disease progression (Bensimon *et al*, 1994; Abe *et al*, 2017). The cornerstone of patient management remains multidisciplinary care, with nutritional and respiratory support (Hardiman *et al*, 2017; Masrori & Van Damme, 2020). This lack of effective treatments is in part attributed to the genetic heterogeneity of ALS, as mutations in over 20 genes are linked to the disease (Al‐Chalabi *et al*, 2017). In 10% of patients, there is a clear family history and about 60–70% of familial cases can be explained by gene mutations in “*chromosome 9 open reading frame 72”* (*C9ORF72*), “*fused in sarcoma”* (*FUS*), “*superoxide dismutase 1”* (*SOD1*), or “*TAR DNA binding protein”* (*TARDBP*) gene. The remaining 90% of patients have no affected relatives and are classified as sporadic ALS. In the majority of sporadic patients, the cause remains unknown. Yet, cytoplasmic inclusions containing TAR DNA‐binding protein 43 kDa (TDP‐43) are the pathological hallmark in 98% of all patients (Neumann *et al*, 2006; French *et al*, 2019) suggesting that TDP‐43 dysfunction is implicated in both familial and sporadic forms of the disease. Additionally, TDP‐43 inclusions are also observed in up to 50% of patients with frontotemporal dementia (FTD). Frontotemporal dementia is a neurodegenerative disorder characterized by neuronal loss in the frontal and anterior temporal lobes, which presents with behavioral changes, executive dysfunction, and/or language problems (Neary *et al*, 2005). Mutations in *TARDBP*, the gene encoding the TDP‐43 protein are a cause of familial ALS, and in rare cases also of ALS‐FTD or FTD (Kabashi *et al*, 2008; Sreedharan *et al*, 2008; Van Deerlin *et al*, 2008; Lemmens *et al*, 2009; Borroni *et al*, 2009; Caroppo *et al*, 2016). TDP‐43 aggregation has also been seen in the brain of some Alzheimer’s disease (AD) patients, with a clinical indistinguishable AD phenotype (Chang *et al*, 2016). Furthermore, TDP‐43 is known to interact with Aβ plaques, resulting in toxic oligomers formation, which may be implicated in AD pathogenesis (Josephs *et al*, 2014; Davis *et al*, 2017). Recently, TDP‐43 pathology has also been identified in age‐related encephalopathies and is found in about 25% of individuals above the age of 80 years (Nelson *et al*, 2019). These findings have expanded the spectrum of TDP‐43‐associated disorders and highlight the importance of understanding the molecular mechanisms underlying these pathologies.

TDP‐43 is a RNA‐ and DNA‐binding protein that primarily resides in the nucleus, but is known to shuttle to the cytosol (Ayala *et al*, 2008; Winton *et al*, 2008). TDP‐43 plays a role in multiple cellular processes including transcriptional repression, pre‐mRNA splicing, mRNA stability, microRNA biogenesis, RNA transport, and translational regulation (Buratti *et al*, 2010; Polymenidou *et al*, 2011). As TDP‐43 regulates the splicing and stability of thousands of mRNA transcripts, it regulates diverse cellular processes (Cohen *et al*, 2011; Tsuiji *et al*, 2011; Butti & Patten, 2019). Since TDP‐43 is inherently prone to aggregation (Johnson *et al*, 2009; Kumar *et al*, 2019), cells need to maintain a tight regulation of its levels to preserve its function and prevent aggregation. To this end, TDP‐43 binds to and regulates the stability of its own mRNA transcript, hereby generating an auto‐regulatory feedback loop (Ayala *et al*, 2011; Da Cruz & Cleveland, 2011; Eréndira Avendaño‐Vázquez *et al*, 2012; White *et al*, 2018; Weskamp & Barmada, 2018).

In most ALS and FTD cases, TDP‐43 is the core component of the cytoplasmic inclusions found in neuronal and glial cells. These inclusions are seen in cells that also display nuclear depletion of TDP‐43 (Arai *et al*, 2006; Neumann *et al*, 2006; Winton *et al*, 2008). Therefore, two non‐mutually exclusive disease mechanisms have been proposed, being nuclear loss‐of‐function and cytoplasmic gain‐of‐function. Nuclear loss of TDP‐43 function originates from its nuclear depletion, hence, disturbing its pleiotropic role in RNA metabolism (Highley *et al*, 2014). On the other hand, the cytoplasmic accumulation and aggregation of TDP‐43, resulting in sequestration of various proteins and overloading of the protein degradation machinery, present a potential toxic gain‐of‐function mechanism (Lin *et al*, 2017). Indeed, several animal models have shown that both knockdown and overexpression of TDP‐43 are toxic (Gendron & Petrucelli, 2011), suggesting that both disease mechanisms may be at play.

So far, no treatment directed toward TDP‐43 pathology has been developed, mainly due to lack of ALS disease models that reliably replicate pathological hallmarks of the disease. Induced pluripotent stem cells (iPSCs) provide a new opportunity to model human disease starting from patient material, allowing us to investigate the effect of disease‐causing mutations in their physiological context. In this study, we used iPSC‐derived motor neurons to understand the basic disease mechanisms caused by mutations in the *TARDBP* gene. We discovered that iPSC‐derived motor neurons recapitulate key pathological hallmarks of TDP‐43 proteinopathy. Using endogenously wild‐type and mutant TDP‐43 tagged cell lines, we showed the mutation‐specific contribution to the disease phenotypes. Moreover, we observed that mutant TDP‐43 causes axonal transport defects as observed previously in models of *SOD1* (Kiskinis *et al*, 2014), *C9ORF72* (Fumagalli *et al*, 2019; Abo‐rady *et al*, 2020), and *FUS* (Guo *et al*, 2017), further implicating this mechanism in the pathogenesis of ALS/FTD. The use of allele‐specific tagged cell lines allowed us to directly link TDP‐43 pathology with axonal transport defects in patient iPSC‐derived motor neurons. Lastly, we found that both the pathological hallmarks and axonal transport defects can be rescued by genetic correction of the mutation using CRISPR/Cas9 or by treatment with a selective HDAC6 inhibitor. These findings underline the value of iPSC‐derived motor neurons as a preclinical model for ALS/FTD and highlight the therapeutic potential of HDAC6 inhibitors for TDP‐43 proteinopathies.

## Results

### Mutant TDP‐43 iPSCs differentiate normally into motor neurons

Starting from patient‐derived fibroblasts, we generated iPSCs from three ALS patients carrying heterozygous point mutations in *TARDBP*: one with a G287S mutation, one with a N390S mutation, and one carrying both an A382T mutation and a *C9ORF72* repeat expansion (Appendix Table S1). Together with three control lines, these patient‐derived iPSCs were differentiated into motor neurons using a previously described protocol (Maury *et al*, 2015; Guo *et al*, 2017; Fumagalli *et al*, 2019; Vandoorne *et al*, 2019) (Appendix Fig S1A). Immunofluorescent staining was performed to characterize the differentiated neurons (Appendix Fig S1B). At day 38 of differentiation, neurons stained positive for pan‐neuronal markers (*i.e*., synapsin and TUJ1) as well as for specific motor neuron markers (*i.e.*, choline acetyltransferase (CHAT), SMI‐32, and ISL1). Quantification of CHAT, SMI‐32, and ISL1‐positive cells demonstrated a high motor neuron differentiation efficiency ranging between 70 and 85%, without significant differences between mutant TDP‐43 and healthy control cell lines (Appendix Fig S1C–E). This was confirmed by RNA sequencing revealing similar CHAT, SMI‐32, and ISL1 expression levels in mutant TDP‐43 and healthy control iPSC‐derived motor neurons, with low expression of non‐neuronal markers (Appendix Fig S2A). Next, electrophysiological activity was analyzed to investigate the functionality of the differentiated motor neurons. Motor neurons derived from both controls and patients displayed properties of mature motor neurons, *i.e*., the presence of voltage‐gated Na^+^ and K^+^ currents as well as the ability to fire spontaneous and evoked action potentials (Appendix Fig S2B–D). Altogether, we generated functional mutant TDP‐43 iPSC‐derived motor neurons with a high efficiency.

### Mutant TDP‐43 motor neurons accumulate insoluble TDP‐43 species containing high levels of C‐terminal TDP‐43 fragments

**Figure 1:**
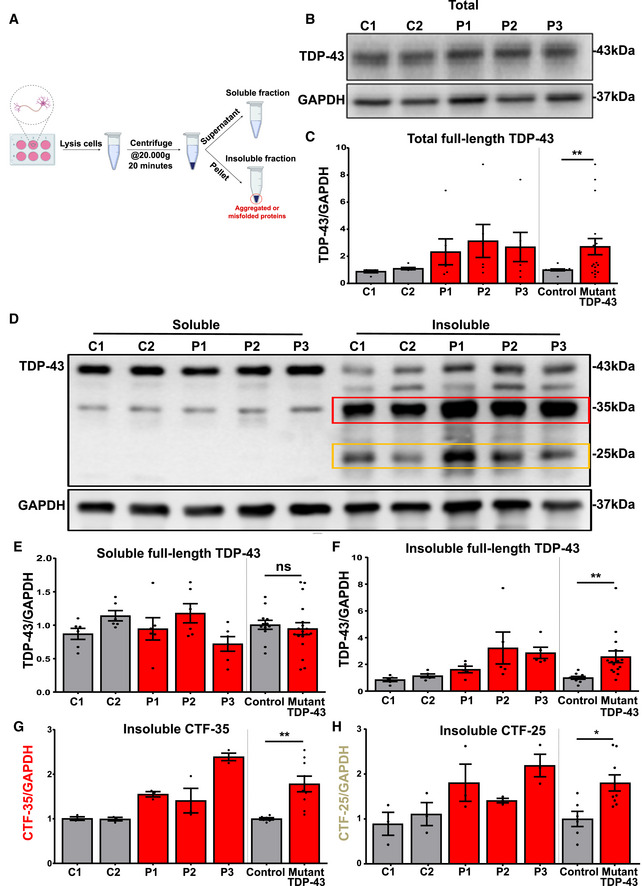
Increased levels of insoluble TDP‐43 and C‐terminal TDP‐43 fragments in mutant TDP‐43 motor neurons AScheme representing the workflow of fractionation into soluble and insoluble fractions.B, C(B) Representative Western blot showing total TDP‐43 levels (unfractionated), quantification in (C), ratio paired *t*‐test.DWestern blot analysis of soluble and insoluble fractions (Red and yellow rectangles highlight CTF‐35 and CTF‐25, respectively, in the insoluble fraction).EQuantification of soluble full‐length TDP‐43, ratio paired *t*‐test.FQuantification of insoluble full‐length TDP‐43, ratio paired *t*‐test.GGraph contains quantification of insoluble c‐terminal fragment of 35 kDa (CTF‐35), ratio paired *t*‐test.HGraph indicates quantification of insoluble c‐terminal fragment of 25 kDa (CTF‐25), ratio paired *t*‐test. Data information: Data are shown as mean ± SEM, *P < 0.05, **P < 0.01, ns: not significant. Each dot represents an independent differentiation in all panels: Data obtained from five‐six independent differentiations.

While TDP‐43 aggregation is a key hallmark of ALS/FTD pathology, few studies have studied this in detail in iPSC‐derived cell models. We did not observe any overt aggregates of TDP‐43 using confocal microscopy. Previously, it has been reported that iPSC‐derived motor neurons carrying VCP mutations demonstrate mislocalized TDP‐43 (Hall *et al*, 2017), while those possessing TDP‐43 mutations show enhanced levels of insoluble TDP‐43 (Bilican *et al*, 2012). Therefore, we performed fractionation experiments on differentiated motor neurons as it is highlighted in the scheme of figure 1 (Fig 1A), to quantify TDP‐43 levels. First, Western blot analysis indicated higher levels of total TDP‐43 protein in the mutant motor neurons compared to controls (Fig 1B and C). This finding is in line with a perturbed loss of TDP‐43’s autoregulation or increased stability and has been observed in other models (Li *et al*, 2010; Wegorzewska & Baloh, 2011; Lin *et al*, 2017; Beel *et al*, 2018). Second, mutant TDP‐43 motor neurons had similar levels of soluble TDP‐43 compared to control motor neurons (Fig 1D and E). However, the levels of insoluble TDP‐43 were elevated in mutant lines (Fig 1D and F). In addition, this insoluble fraction contained C‐terminal TDP‐43 fragments, *i.e*., CTF‐35 kDa and CTF‐25 kDa fragments, which were significantly elevated in mutant *versus* control motor neurons (Fig 1D, G, H). These findings show that our iPSC model displayed TDP‐43 dysfunctions commonly associated with ALS/FTD, exhibiting elevated insolubility and C‐terminal cleavage of TDP‐43 (Berning & Walker, 2019). Although we did not observe TDP‐43 positive inclusions as seen in post mortem material, the accumulation of insoluble full‐length and cleaved TDP‐43 may be an early step in the sequence of events of a TDP‐43 proteinopathy.

### Mutant TDP‐43 motor neurons exhibit cytoplasmic mislocalization and phosphorylation of TDP‐43

**Figure 2:**
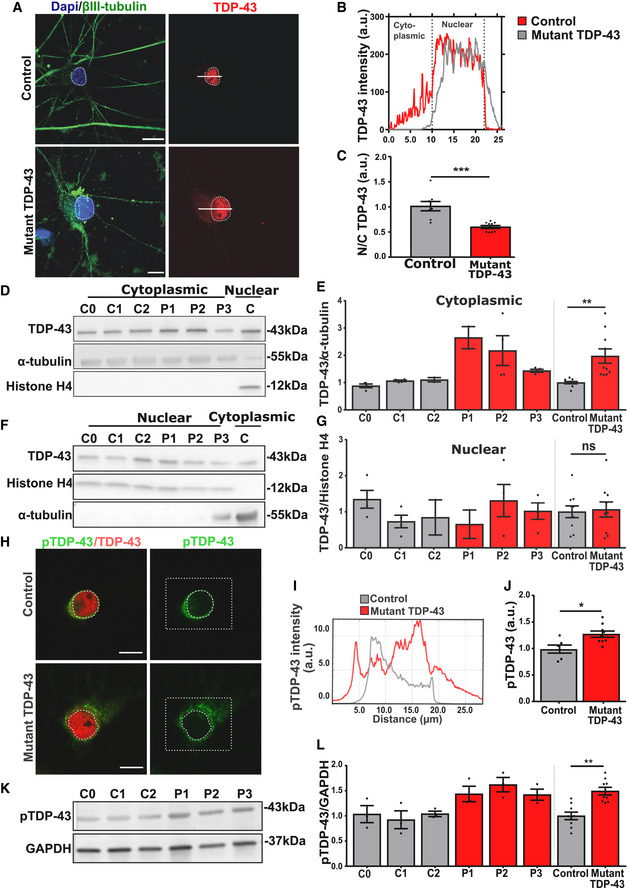
Mutant TDP‐43 motor neurons exhibit TDP‐43 cytoplasmic mislocalization and increased phosphorylation AImmunofluorescent analysis of TDP‐43 in control and mutant TDP‐43 iPSC‐derived motor neurons. Upper images represent control and lower mutant TDP‐43 motor neurons. Presented immunofluorescent images of control (C2) and mutant TDP‐43 (P2), Scale bar: 20 µm.BCorresponding profile intensity plot of panel A.CQuantification of the nucleo‐cytoplasmic ratio (N/C) of TDP‐43 fluorescent intensity of pooled control and pooled mutant motor neurons, unpaired Mann–Whitney test.D, E(D) Western blot analysis of cytoplasmic fraction with a positive nuclear control (C) confirming the fractionation purity, quantification in (E), ratio paired *t*‐test.F, G(F) Western blot analysis of nuclear fraction with a positive cytoplasmic control (C) confirming the fractionation purity, quantification in (G), ratio paired *t*‐test.H–JImmunofluorescent analysis showing fluorescence intensity of phosphorylated TDP‐43 (pTDP‐43). Scale bar: 20 µm. Presented immunofluorescent images of control (C2) and mutant TDP‐43 (P2). (I) represents its corresponding profile intensity plot of the selected (square) region. Scale bar: 10 µm. (J) quantification pooled control (C0 = 10, C1 = 10, and C2 = 10) *n* = 30 and pooled mutant TDP‐43 (P1 = 10, P2 = 10, and P3 = 10) *n* = 30 cells from each differentiation. Unpaired Mann–Whitney test.K, L(K) Western blot analysis showing pTDP‐43, quantification in (L), ratio paired *t*‐test. Data information: Data are shown as mean ± SEM, *P < 0.05, **P < 0.01, ***P < 0.001, ns: not significant. Each dot represents an independent differentiation in all panels: Data obtained from three independent differentiations.

Nuclear depletion of TDP‐43 and its accumulation in cytoplasmic inclusions is the most common pathological hallmark of ALS/FTD (Neumann *et al*, 2006). We therefore studied the subcellular distribution of TDP‐43 in mutant TDP‐43 iPSC‐derived motor neurons. Immunohistochemistry revealed cytoplasmic mislocalization of TDP‐43 in mutant TDP‐43 motor neurons, in comparison with control iPSC‐derived motor neurons (Fig 2A). Accordingly, there was a decreased TDP‐43 nuclear/cytoplasmic ratio in mutant TDP‐43 motor neurons compared to controls (Fig 2B and C). Using Western blot analysis of the cytoplasmic and nuclear fractions of iPSC‐derived motor neurons, we confirmed that mutant TDP‐43 motor neurons contained higher cytosolic TDP‐43 levels (Fig 2D and E) compared to control motor neurons, whereas nuclear TDP‐43 levels were unaltered (Fig 2F and G). Altogether, this suggests that mutant TDP‐43 causes an imbalance of the normal nucleo‐cytoplasmic distribution of TDP‐43.

Besides its translocation to the cytoplasm, TDP‐43 is also known to be abnormally phosphorylated in patients (Neumann *et al*, 2007; Hasegawa *et al*, 2008; Igaz *et al*, 2008). First, we found abundant staining in the soma region of mutant TDP‐43, using phospho‐specific antibodies for TDP‐43 (Fig 2H–J). Second, we found a disease‐specific accumulation of phosphorylated TDP‐43 using Western blot analysis (Fig 2K and L).

### Only mutant TDP‐43 is mislocalized and abnormally phosphorylated in mutant TDP‐43 motor neurons

**Figure 3:**
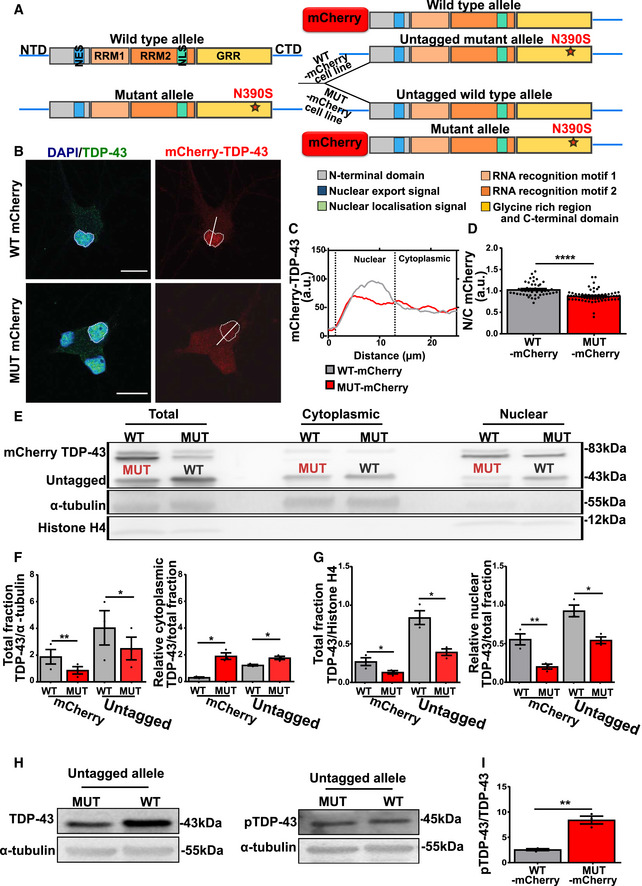
Mutant TDP‐43 undergoes cytoplasmic mislocalization and increased phosphorylation in mutant TDP‐43 motor neurons AScheme representing the two cell lines generated from the mutant TDP‐43 iPSCs by either endogenously tagging the WT TDP‐43 allele or the mutant TDP‐43 allele with mCherry.B, C(B) Immunofluorescent analysis with in (C) its corresponding intensity plot showing cytoplasmic mislocalization of the MUT‐mCherry TDP‐43 in motor neurons, but not in WT‐mCherry TDP‐43. Scale bar: 20 µm.DQuantification of nucleo‐cytoplasmic (N/C) intensity ratio based on immunohistochemistry of WT‐mCherry (*n* = 45) and MUT‐mCherry (*n* = 63) tagged TDP‐43. Each dot represents one analyzed cell, unpaired Mann–Whitney test.E, F(E) Western blots showing nucleo‐cytoplasmic fractionation of WT‐mCherry and MUT‐mCherry iPSC‐derived motor neurons with (F) (left panel) Quantification of total mCherry and total untagged TDP‐43 on α‐tubulin and (right panel) relative cytoplasmic mCherry and untagged TDP‐43 on total mCherry and total untagged TDP‐43, respectively, ratio paired *t*‐test.G(left panel) Quantification of total mCherry and total untagged TDP‐43 on Histone H4 and (right panel) relative nuclear mCherry and untagged TDP‐43 on total mCherry and total untagged TDP‐43, respectively, ratio paired *t*‐test.H, I(H) Western blot showing untagged TDP‐43 (left panel) and phosphorylation state of the untagged allele (right panel) in WT‐ and MUT‐mCherry motor neurons and in (I) ratio quantification of normalized pTDP43/normalized TDP‐43 (because of almost the same molecular weight two different blots were used), ratio paired *t*‐test. Data information: Data are shown as mean ± SEM, *P < 0.05, **P < 0.01, ****P < 0.0001. Each dot represents an independent differentiation in (F, G, and I) panels: Data obtained from three independent differentiations.

To investigate the causal relationships between mutations in the *TARDBP* gene and the observed TDP‐43 pathology in mutant TDP‐43 motor neurons, we made use of CRISPR‐Cas9 genome technology to tag the wild‐type (WT‐mCherry) or the mutant (MUT‐mCherry) allele of the *TARDBP* gene with the red fluorescent protein mCherry. We hereby generated two distinct mutant TDP‐43 patient cell lines, one with the mutant and the other with the WT allele tagged, starting from a mutant TDP‐43 iPSC line (P2 (N390S)) (Fig 3A and Appendix Fig S3A–C). We additionally performed a genomic hybridization (CGH) array, to check for any differences in copy number variations which may occur because of CRISPR‐Cas9 genome technology in WT‐mCherry (Appendix Fig S3D left panel) and MUT‐mCherry (Appendix Fig S3D right panel). These tagged iPSC lines were differentiated into motor neurons, with no differences observed at the levels of differentiation efficiency by evaluating immunohistochemistry analysis (Appendix Fig S3E–H). However, using confocal microscopy MUT‐mCherry TDP‐43 displayed an abnormal nucleo‐cytoplasmic distribution compared to the WT‐mCherry TDP‐43 protein (Fig 3B–D), which had a predominant nuclear localization comparable to the signal of TDP‐43 in control lines. Although the total levels of the mutant protein were lower, the cytoplasmic accumulation could be confirmed using Western blot (Fig 3E). After nucleo‐cytoplasmic fractionation, we observed higher cytosolic (Fig 3F right panel) and lower nuclear (Fig 3G right panel) levels of mutant mCherry TDP‐43.

The phospho‐TDP‐43 antibody (Fig 2H–L) did not recognize the mCherry‐tagged TDP‐43 bands. In order to study the phosphorylation levels, we quantified the untagged TDP‐43 levels in MUT‐mCherry and WT‐mCherry TDP‐43 lines. A much larger proportion of the mutant untagged TDP‐43 was phosphorylated compared to the wild‐type TDP‐43 protein (Fig 3H and I). Our data show that specifically mutant TDP‐43 is mislocalized and phosphorylated in comparison with wild‐type TDP‐43 in human iPSC‐derived motor neurons.

### Mutant TDP43 iPSC‐derived motor neurons display mitochondrial transport defects

**Figure 4:**
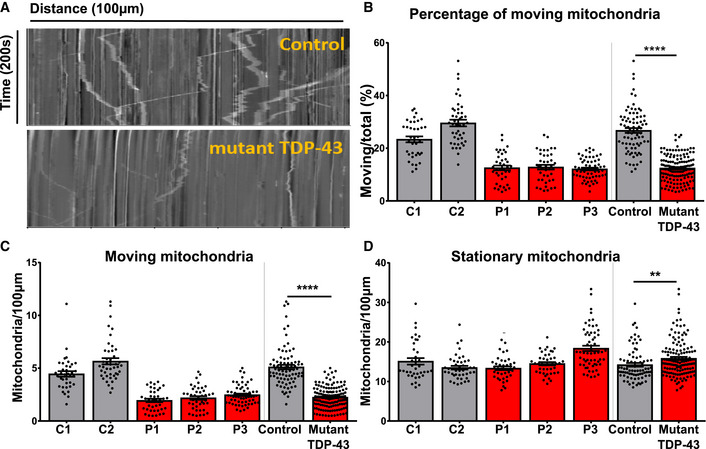
Mutant TDP‐43 motor neurons display mitochondrial transport defects Representative kymographs from control and mutant TDP‐43 iPSC‐derived motor neurons. The x‐axis represents distance in µm while on the y‐axis time in seconds is revealed. Stationary mitochondria are visible as straight vertical lines, while moving mitochondria are depicted as skewed lines.Quantification of percentage of moving mitochondria.Quantification of the absolute number of moving mitochondria.Quantification of stationary mitochondria normalized to neurite length 100 µm. Data information: Data are shown as mean ± SEM, **P < 0.01, ****P < 0.0001. For panels (B, C, and D): Each dot represents one neurite for control (n = 81) and mutant TDP‐43 (n = 141) neurites. Mann–Whitney test in all panels. Data combined from three independent differentiations.

In order to explore if the TDP‐43 proteinopathy we observed was associated with neuronal defects, we studied axonal transport, which is essential for normal neuronal functioning (D’Ydewalle *et al*, 2011; Shen *et al*, 2016; Guo *et al*, 2017). Defects in axonal transport are an important disease mechanism in ALS/FTD, with several ALS‐associated genes being implicated in axonal transport, and axonal transport defects being observed in various disease models (Guo *et al*, 2019). We treated mutant TDP‐43 and healthy control iPSC‐derived motor neurons with MitoTracker‐RED and performed live cell imaging. Using kymographs, we assessed the motility of the mitochondria along the neurites (Fig 4A). We found a significant decrease in the percentage and number of moving mitochondria (Fig 4B and C) and an increase in the number of stationary mitochondria (Fig 4D) in mutant TDP‐43 motor neurons compared to controls. Importantly, the total number of mitochondria was unaltered. Taken together, these data are in line with a previous study showing mutant TDP‐43 iPSC‐derived motor neurons to display a defect in mitochondrial transport along the neurites (Kreiter *et al*, 2018).

### CRISPR/Cas9‐mediated gene editing reverses mutant TDP‐43‐associated phenotypes

**Figure 5:**
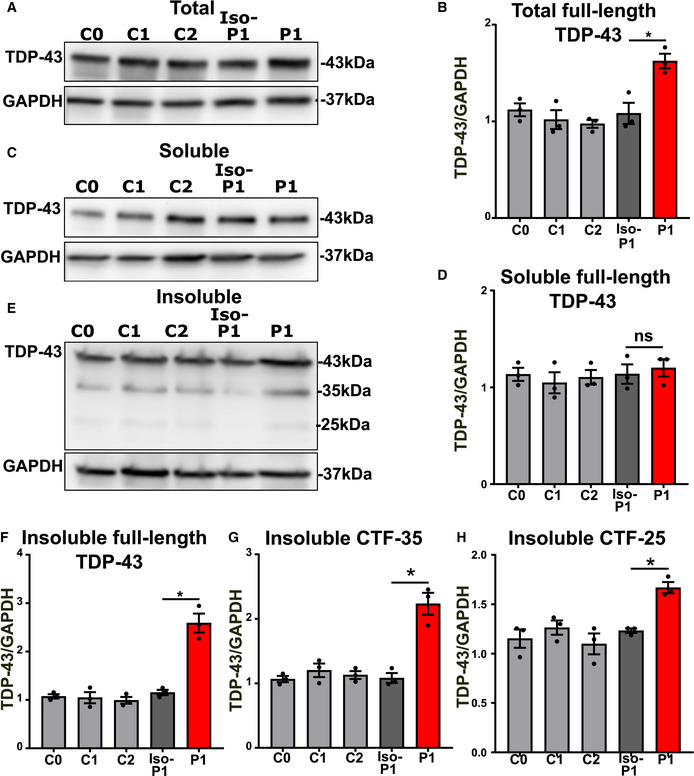
CRISPR/Cas9‐mediated gene editing reverse total and insoluble TDP‐43 AWestern blot analysis of total fraction of TDP‐43 levels in healthy controls (C0, C1, and C2), isogenic P1 (Iso‐P1), and mutant P1 (P1) motor neurons.BQuantification of total full‐length TDP‐43 levels, ratio paired *t*‐test.C, D(C) Western blot of soluble fraction and (D) quantification of soluble full‐length TDP‐43, ratio paired *t*‐test.E, F(E) Western blot of insoluble fraction and (F) quantification of insoluble full‐length TDP‐43, ratio paired *t*‐test.G, H(G) quantification of insoluble CTF‐35, ratio paired *t*‐test and (H) quantification of insoluble CTF‐25, ratio paired *t*‐test. Data information: Data are shown as mean ± SEM. *P < 0.05, ns: not significant. Each dot represents an independent differentiation in all panels: Data combined from three independent differentiations.

To confirm that the observed phenotypes were directly linked to the presence of mutant TDP‐43, we edited the genetic defect. We corrected the G287S mutation in one of the ALS lines (P2) resulting in an isogenic control line (G287G) (Appendix Fig S4A–D). To investigate whether correction of the mutation reversed mutant TDP‐43 associated phenotypes, we performed imaging and Western blot analyses. Total TDP‐43 levels were decreased in the isogenic control motor neurons to normal levels (Fig 5A and B). By studying the soluble and insoluble fractions, we found that while the soluble TDP‐43 levels remained similar (Fig 5C and D), the genetic correction restored the accumulation of insoluble TDP‐43 levels (Fig 5E and F), and of the C‐terminal fragments, CTF‐35 (Fig 5E and G) and CTF‐25 (Fig 5E and H) observed in the mutant TDP‐43 motor neurons compared to control.

**Figure 6:**
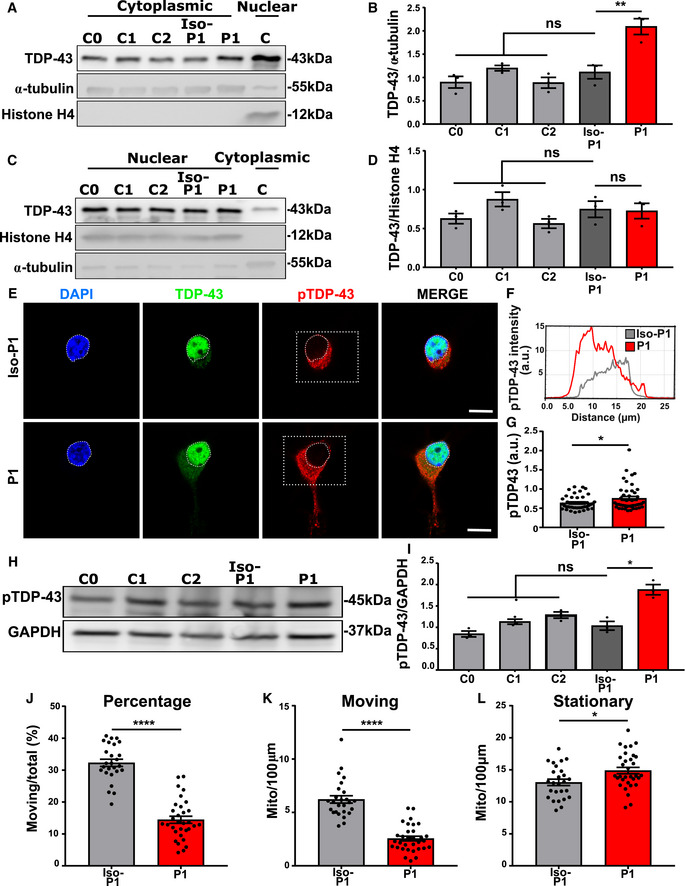
CRISPR/Cas9‐mediated gene editing reverses mutant TDP‐43‐associated phenotypes AWestern blot analysis of cytoplasmic TDP‐43 protein levels in healthy controls (C0, C1, and C2), isogenic P1 (Iso‐P1), and mutant P1 (P1) motor neurons.BQuantification of cytoplasmic TDP‐43 levels, each dot represents an independent differentiation, ratio paired *t*‐test.C, D(C) Western blot analysis of nuclear TDP‐43 and (D) quantification of nuclear TDP‐43 levels, each dot represents an independent differentiation, ratio paired *t*‐test.E, F(E) Immunofluorescent analysis showing fluorescence intensity of pTDP‐43 in motor neurons. Scale bar: 10 µm and its perspective intensity plot in (F).GQuantification of (E), each dot represents one analyzed cell, unpaired Mann–Whitney test.H, I(H) Western blot analysis showing pTDP‐43 levels in healthy controls, Iso‐P1 and P1, quantification in (I), each dot represents one analyzed cell, unpaired Mann–Whitney test.J–LAxonal transport analysis. Quantification of percentage of moving mitochondria (J), number of moving mitochondria (K), and number of stationary mitochondria (L) normalized to neurite length 100 µm. Each dot represents one neurite for Iso‐P1 (*n* = 26) and P1 (*n* = 32) neurites. Unpaired Mann–Whitney test. Data information: Data are shown as mean ± SEM. *P < 0.05, **P < 0.01, ****P < 0.0001, ns: not significant. Data combined from three independent differentiations.

Furthermore, the accumulation of TDP‐43 in the cytoplasmic fraction was also restored by correcting the mutation (Fig 6A and B), while nuclear concentrations were unaffected (Fig 6C and D). In addition, the abnormal phosphorylation of TDP‐43 was abolished in the isogenic motor neurons as seen using immunofluorescent staining (Fig 6E–G) and confirmed by Western blot (Fig 6H and I). Next, we tested the effect of the genetic correction on the axonal transport phenotype. In comparison with mutant TDP‐43 and control motor neurons, the isogenic controls revealed a full rescue of the percentage and number of motile mitochondria (Fig 6J and K) and the number of stationary mitochondria was normal in the isogenic control (Fig 6L). These findings confirm that the observed phenotypes are a direct consequence of the presence of mutant TDP‐43.

### HDAC6 inhibition rescues TDP‐43 pathology in mutant TDP‐43 iPSC‐derived motor neurons

**Figure 7:**
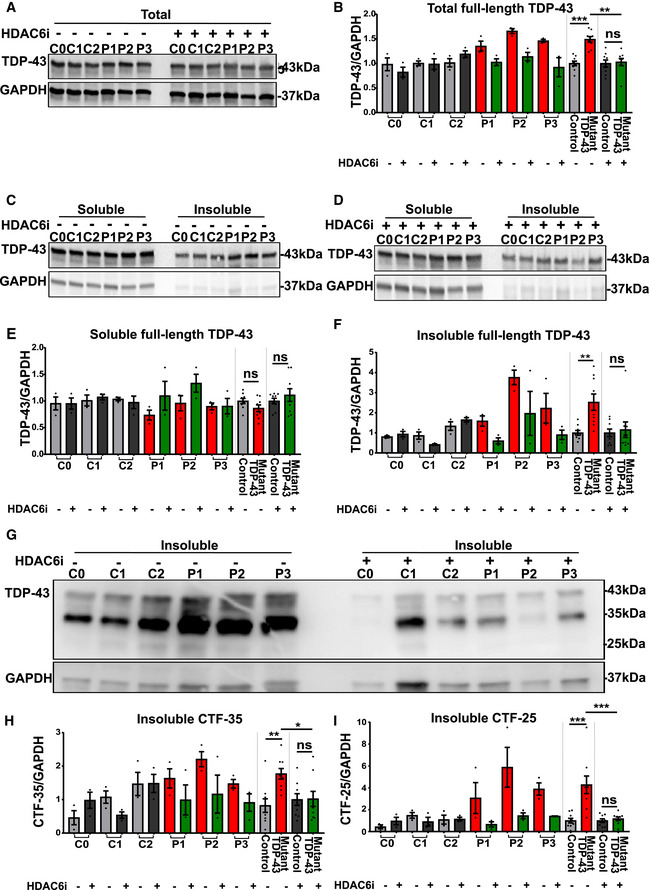
HDAC6 inhibitor reduces total TDP‐43 levels by reducing the insoluble TDP‐43 levels and c‐terminal fragmentations in mutant TDP‐43 iPSC‐derived motor neurons A–D(A) Western blot showing total TDP‐43 levels, quantification in (B), ratio paired *t*‐test. Total cell lysates were fractionated into soluble and insoluble fractions and analyzed by Western blot, representative Western blots of soluble and insoluble fraction without (C) and with (D) HDAC6 inhibitor treatment.E, F(E) Quantification of soluble full‐length TDP‐43 levels, ratio paired *t*‐test and (F) quantification of insoluble full‐length TDP‐43 levels, ratio paired *t*‐test.GWestern blots of insoluble fraction with (right) and without (left) HDAC6 inhibitor treatment.H, IQuantification of insoluble c‐terminal fragment of 35 kDa (CTF‐35; H) and insoluble c‐terminal fragment of 25 kDa (CTF‐25; I), ratio paired *t*‐test. Data information: Data are shown as mean ± SEM, *P < 0.1, **P < 0.01, ***P < 0.001, ns: not significant. Each dot represents an independent differentiation in all panels: Data combined from three independent differentiations.

Previously, a direct interaction between TDP‐43 and histone deacetylase 6 (HDAC6) has been shown to play a role in the sequestration of TDP‐43 in the cytosol (Hebron *et al*, 2013). HDAC6 is known to play a role in the degradation of misfolded proteins and inhibition of HDAC6 can stimulate chaperone expression (Cook *et al*, 2012). Therefore, we evaluated the effect of the selective HDAC6 inhibitor Tubastatin A on the observed TDP‐43 pathologies in mutant TDP‐43 iPSC‐derived motor neurons. We treated iPSC‐derived motor neurons with Tubastatin A for 12 h and assessed TDP‐43 levels using immunofluorescent staining and Western blot. HDAC6 inhibition normalized the elevated TDP‐43 protein levels observed in mutant TDP‐43 lines (Fig 7A and B). Whereas soluble levels remained unaltered (Fig 7C–E), the insoluble levels were significantly reduced (Fig 7C, D, F). In the insoluble fraction, we also observed a strong reduction of the levels of CTF‐35 (Fig 7G and H) and CTF‐25 (Fig 7G and I) to control levels. Tubastatin A treatment did not affect TDP‐43 levels, insolubility, or CTF levels in control lines, suggesting that this treatment specifically reversed defects caused by mutant TDP‐43.

**Figure 8:**
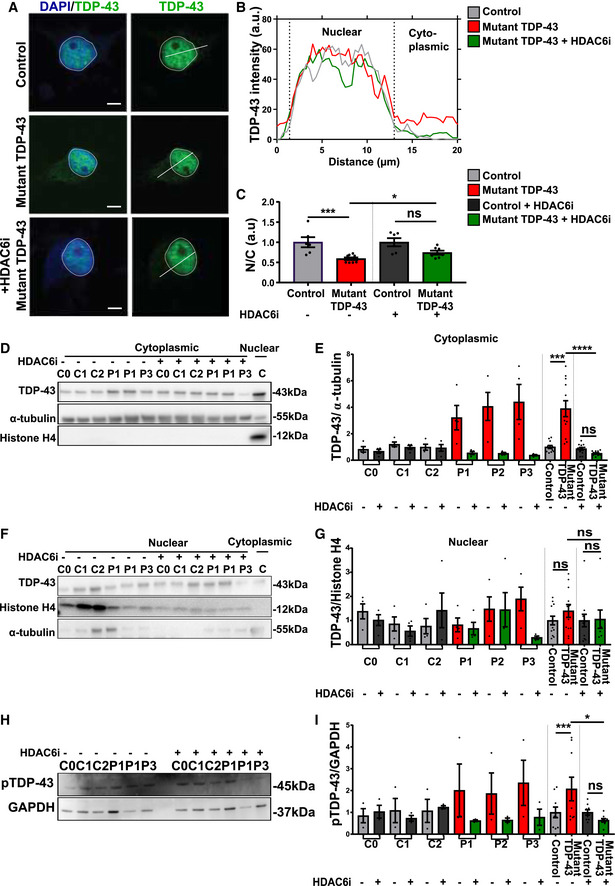
HDAC6 inhibitor restores subcellular mislocalization and abnormal phosphorylation of TDP‐43 in mutant TDP‐43 iPSC‐derived motor neurons AImmunofluorescent analysis of TDP‐43 in control, mutant TDP‐43, and mutant TDP‐43 + HDAC6 inhibitor after 12 h treatment. Presented immunofluorescent images of control (C2) and mutant TDP‐43 (P2). Scale bar: 25 µm.BProfile intensity plot of control, mutant TDP‐43, and mutant TDP‐43 + HDAC6 inhibitor.CQuantification of nucleo‐cytoplasmic ratio (N/C) fluorescent intensity of TDP‐43 in motor neurons, each dot represents one analyzed cell, unpaired Mann–Whitney test.D, E(D) Western blot analysis of cytosolic fraction, quantification in (E), ratio paired *t*‐test.F, G(F) Western blot analysis of nuclear fraction, quantification in (G), ratio paired *t*‐test.H, I(H) Western blot analysis showing pTDP‐43 levels and (I) quantification, ratio paired *t*‐test. Data information: Data are shown as mean ± SEM, *P < 0.05, ***P < 0.001, ****P < 0.0001, ns: not significant. Each dot represents an independent differentiation in (E, G, and I) panels: Data combined from three independent differentiations.

Additionally, HDAC6 inhibition rescued the cytoplasmic mislocalization of TDP‐43 (Fig 8A–G) and restored abnormal phosphorylation (Fig 8H and I) of TDP‐43 to control levels. Similar observations were made in our isogenic pair of mutant TDP‐43 G287S line and its isogenic control G287G (Appendix Fig S5A–I).

**Figure 9:**
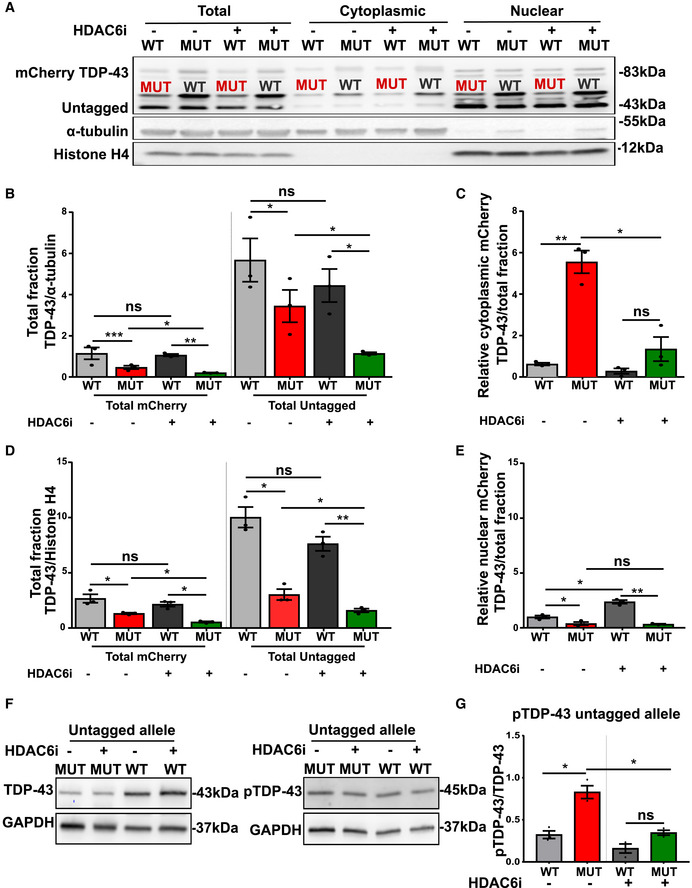
HDAC6 inhibitor reduces cytoplasmic MUT‐mCherry TDP‐43 and enhances nuclear WT‐mCherry TDP‐43 ARepresentative Western blot of total (mCherry and untagged), cytoplasmic TDP‐43 and nuclear levels with and without HDAC6 inhibitor.BQuantification of total mCherry and total untagged relative to α‐tubulin, ratio paired *t*‐test.CQuantification of relative cytoplasmic mCherry TDP‐43 on total fraction, ratio paired *t*‐test.DQuantification of total mCherry and total untagged relative to Histone H4, ratio paired *t*‐test.EQuantification of relative nuclear mCherry TDP‐43 on total fraction, ratio paired *t*‐test.F, G(F) Western blot showing untagged TDP‐43 (left panel) and phosphorylation state of the untagged allele (right panel) in WT‐ and MUT‐mCherry motor neurons and in (G) ratio quantification of normalized pTDP43/normalized TDP‐43 (because of almost the same molecular weight two different blots were used), ratio paired *t*‐test. Data information: Data are shown as mean ± SEM, *P < 0.05, **P < 0.01, ***P < 0.001, ns: not significant. Each dot represents an independent differentiation in all panels: Data combined from three independent differentiations.

To explore the therapeutic effect of Tubastatin A on the allele‐specific subcellular distribution and abnormal phosphorylation of wild‐type and mutant TDP‐43 protein, we used the tagged cell lines. Upon treatment with Tubastatin A, the cytosolic levels of MUT‐mCherry TDP‐43 were reduced (Fig 9A–C), while WT‐mCherry TDP‐43 levels remained similar. On the other hand, nuclear WT‐mCherry TDP‐3 was modestly increased upon treatment with HDAC6i (Fig 9A, D, E), while nuclear MUT‐mCherry TDP‐43 levels were unaffected. By quantifying the ratio of the untagged TDP‐43 and the phosphorylated TDP‐43 band in WT‐mCherry and MUT‐mCherry lines, we confirmed that the proportion of phosphorylated TDP‐43 is higher for mutant versus wild‐type TDP‐43 (Fig 9F and G). Treatment with Tubastatin A did not affect the phosphorylation of wild‐type TDP‐43. However, we found that Tubastatin A restored the abnormal phosphorylation of the untagged mutant TDP‐43 band in WT‐mCherry TDP‐43 lines (Fig 9F and G).

### HDAC6 inhibition rescues mitochondrial transport defects

**Figure 10:**
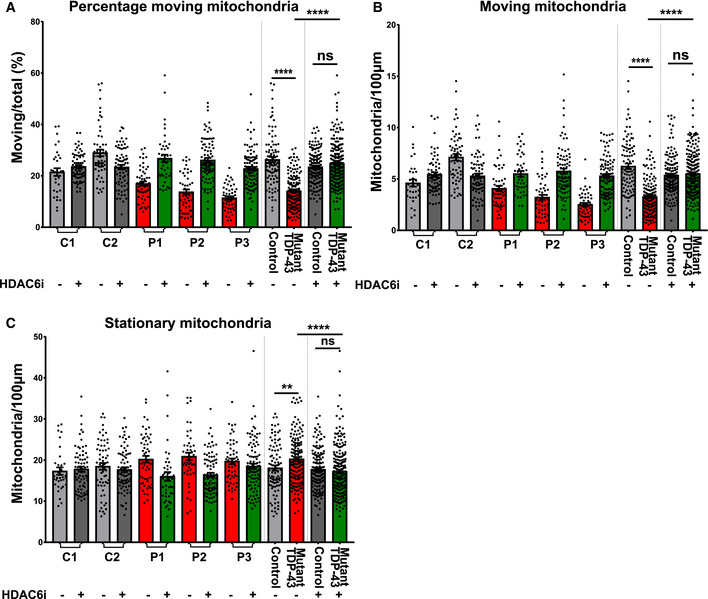
HDAC6 inhibitor rescues mitochondrial transport defects in mutant TDP‐43 iPSC‐derived motor neurons A–C(A) Quantification of percentage of moving mitochondria, (B) quantification of the absolute amount of moving mitochondria, and (C) quantification of the amount of stationary mitochondria normalized to neurite length 100 µm in each quantification. Each dot represents one neurite for control (*n* = 94) versus mutant TDP‐43 (*n* = 159) and control treated with HDAC6 inhibitor (*n* = 145) versus mutant treated with HDAC6 inhibitor (*n* = 235). Mann–Whitney test in all panels: Data were combined from three independent differentiations. Data are shown as mean ± SEM, **P < 0.01, ****P < 0.0001, ns: not significant.

As Tubastatin A could restore TDP‐43 pathology and has been reported to rescue axonal transport deficits in neurodegenerative diseases, including ALS (Guo *et al*, 2017), we explored the effect of Tubastatin A on the axonal transport defects present in the mutant TDP43 iPSC‐derived motor neurons. A 12 h treatment with Tubastatin A restored the motility of axonal mitochondria in mutant TDP‐43 motor neurons compared to controls (Fig 10A and B) and reduced the number of stationary mitochondria (Fig 10C), while leaving the total number of mitochondria unaltered. Finally, we tested the effect of Tubastatin A on TDP‐43 proteinopathy and axonal transport in our mutant TDP‐43 G287S line and its isogenic control G287G (Appendix Fig S5J–L). Tubastatin A treatment reduced the accumulation of insoluble TDP‐43 and ameliorated mitochondrial transport defects in the mutant line, but had no effect on the isogenic control. These findings confirm that Tubastatin A effectively and specifically counteracts mutant TDP‐43‐induced transport defects, while not affecting baseline transport levels.

### TDP‐43 interactome analysis highlights perturbed pathways and therapeutic modulation by HDAC6 inhibition

**Figure 11:**
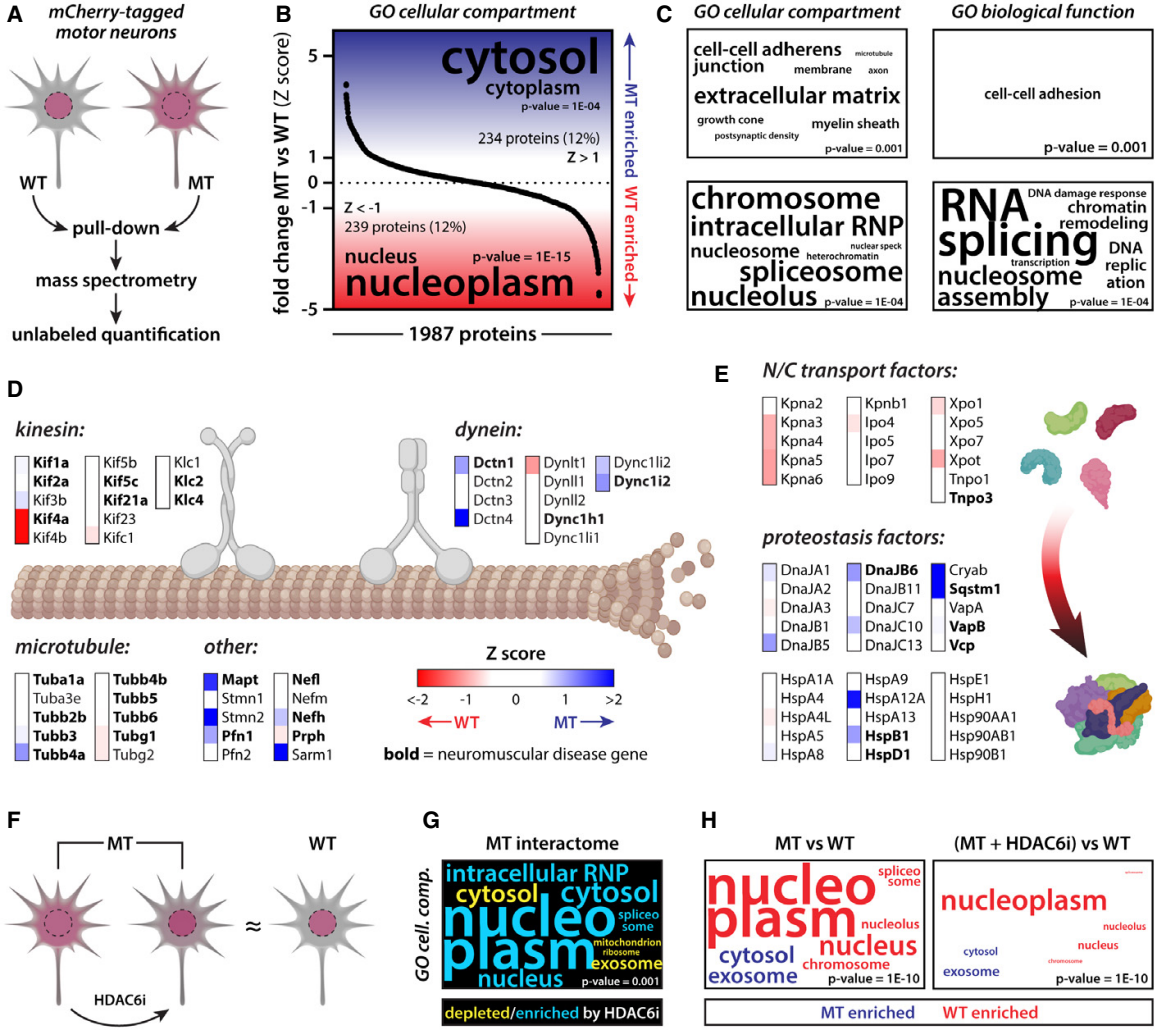
TDP‐43 interactome analysis highlights pathological pathways and therapeutic effect of HDAC6 inhibition AScheme illustrating set‐up of TDP‐43 interactome analysis.BRanked GO enrichment analysis of mutant versus wild‐type TDP‐43 interactors indicates nuclear depletion of mutant TDP‐43.CMore detailed GO analysis further highlights nuclear depletion of mutant TDP‐43 and potential loss‐of‐function mechanisms.DSeveral axonal transport factors differentially interact with mutant versus wild‐type TDP‐43.EThe mutant TDP‐43 interactome is depleted for nuclear transport factors and enriched for proteostasis factors, in line with its observed nuclear depletion and cytoplasmic pathological alterations.FScheme illustrating the effect of HDAC6 inhibition on mutant TDP‐43 phenotypes.G, HRanked GO enrichment analysis highlights the effect of HDAC6 inhibition on the mutant TDP‐43 interactome, in line with its restored nuclear localization.

To get a better understanding on the molecular pathways involved in the observed mutant TDP‐43 phenotypes, we performed differential interactome analysis of mutant versus wild‐type TDP‐43 (Fig 11). The mCherry‐tagged mutant and wild‐type TDP‐43 were pulled down from motor neuron lysates and subjected to unlabeled quantitative mass spectrometry (Fig 11A). We detected just under 2,000 potential interactors in all replicates and conditions (Appendix Table S2). Ranked gene ontology (GO) analysis of our dataset indicated that the mutant TDP‐43 interactome was enriched for cytoplasmic proteins, whereas wild‐type TDP‐43 was enriched for nuclear interaction partners (Fig 11B and C). These findings are in line with the observed nuclear depletion and cytoplasmic mislocalization of mutant TDP‐43 in the motor neuron cultures. Functional GO terms that were associated with enriched wild‐type interactors centered around RNA splicing and other nuclear processes, suggesting that these processes may be altered by TDP‐43 mutations and its nuclear depletion, as indeed others have previously shown (Arnold *et al*, 2013; De Conti *et al*, 2015; Tan *et al*, 2016).

Zooming in on axonal transport, we noticed differential interactions between mutant and wild‐type TDP‐43 with key players in this process (Fig 11D). As several axonal transport factors are genetically linked to neuromuscular disease, these findings suggest that also mutant TDP‐43 may affect this process, confirming our phenotypic observations and tying TDP‐43 dysregulation directly into this important neurodegenerative pathway. Interestingly, Western blot analyses showed that some mutant TDP‐43 interactors involved in microtubule‐based transport factors (DCTN1, TUBA4A, and MAPT) showed increased protein levels in patient lines (Appendix Fig S6A–D). Moreover, while the levels of these interaction partners were similar in the soluble fraction (Appendix Fig S6E–H), they were elevated in the insoluble protein fraction together with TDP‐43 (Appendix Fig S6I–L). These findings indicate that indeed TDP‐43 proteinopathy may directly affect the behavior of key axonal transport factors. Additionally, one of the top mutant interactors (rank 2) was stathmin‐2 (Appendix Table S2), whose expression is regulated by TDP‐43 and whose dysregulation has been shown to alter axonal function (Klim *et al*, 2019; Melamed *et al*, 2019). Besides alterations in axonal transport factors, mutant TDP‐43 was depleted for interactions with nuclear transport factors, which mirrors our observation of its nuclear depletion (Fig 11E). In line with our findings that TDP‐43 demonstrates pathological alterations, we did observe increased interactions of the mutant protein with proteostasis factors (Fig 11E), some of which have been directly implicated in ALS/FTD (e.g., Sqstm1 or p62).

Since Tubastatin A treatment was able to reverse mutant TDP‐43 phenotypes in our motor neuron cultures (Fig 11F), we tested whether it would similarly affect the TDP‐43 interactome. Indeed, HDAC6 inhibition did enrich for nuclear interaction partners in the mutant TDP‐43 interactome and was able to partially normalize the differential mutant versus wild‐type interactome, in line with its restored nuclear localization in cells (Fig 11G and H). These mass spectrometry findings illustrate that TDP‐43 mutations alter its subcellular interactome and that this is likely linked to its dysfunction, yet can be targeted by therapeutic intervention.

## Discussion

In this study, we used patient‐derived motor neurons to study early pathogenic mechanisms induced by mutations in *TARDBP*. Both mutant TDP‐43 and healthy control iPSCs differentiated into motor neurons with similar efficiency and these motor neurons were apparently healthy, without prominent cell death (Appendix Fig S7A). However, early signs of TDP‐43 pathology were seen in motor neurons expressing mutant TDP‐43. These included accumulation of insoluble TDP‐43 levels, elevated levels of CTFs of full‐length TDP‐43 in the insoluble fraction, accumulation of TDP‐43 in the cytoplasm and increased cytoplasmic phosphorylated TDP‐43 in iPSC‐derived motor neurons from *TARDBP* mutation carriers.

TDP‐43 is intrinsically prone to aggregation and ALS‐linked *TARDBP* mutations further increase its tendency to form insoluble aggregated structures (Neumann *et al*, 2006; Bilican *et al*, 2012; Seminary *et al*, 2018). How alterations in TDP‐43 lead to motor neuron degeneration still remains enigmatic. One possible explanation could be the loss‐of‐function hypothesis in the nucleus, which is supported by the fact that cytoplasmic accumulation of TDP‐43 could result in a reduction of functional nuclear TDP‐43, leading to dysregulation of various important RNA processes as previously reported (Freibaum *et al*, 2010). On the other hand, the gain‐of‐function hypothesis focuses on the cytoplasmic role of TDP‐43. TDP‐43 is known to interact with numerous proteins and RNA in the cytosol and cytosolic aggregation or aberrant TDP‐43 function could induce cellular toxicity and cell death (Barmada *et al*, 2010). Our results clearly show the presence of accumulated cytoplasmic insoluble TDP‐43, which could result in cytoplasmic TDP‐43 dysfunction and sequestration of important TDP‐43 interacting proteins. In line with other reports (Burkhardt *et al*, 2013; Arnold *et al*, 2013; Sun *et al*, 2018), we did not observe any clear TDP‐43 inclusions. However, the observed TDP‐43 proteinopathy was accompanied by neuronal dysfunction suggesting that the formation of large fibrillary or amorphous assemblies, as observed in post mortem tissue, are not required to induce cell toxicity and may represent an end‐stage feature of the disease. In addition, we also evaluated RNA levels between control and mutant TDP‐43 iPSC‐derived motor neurons, which shows no differences at RNA level (Appendix Fig S7B). Using the RNA transcriptome data, we looked at the expression of TDP‐43 transcripts with and without the mutation, but both alleles are equally transcribed (Appendix Fig S2B), suggesting that the accumulation of TDP‐43 protein is caused by a longer half‐life and/or reduced turnover of the protein.

To elucidate the contribution of mutant TDP‐43 to the observed phenotypes, we made use of the allele‐specific tagged iPSC lines. By making a distinction between wild‐type‐ and mutant‐tagged motor neurons, we were able to demonstrate in patient‐related cells that specifically the mutant TDP‐43 induces the cytoplasmic accumulation and enhanced phosphorylation of TDP‐43. Wild‐type TDP‐43 was not clearly mislocalized, suggesting that co‐seeding of wild‐type and mutant TDP‐43 is not contributing to the initial steps of TDP‐43 pathology. Additionally, this approach allowed us to perform mutation‐specific interactomics. The presence of a mutation in TDP‐43 did not only alter its localization, but also its interactome. Further research aiming at a better understanding of these two fundamental aspects of TDP‐43 biology will point at important disease pathways.

At the functional level, the changes in TDP‐43 biology were accompanied by axonal transport deficits. Such transport problems have been implicated in ALS before. Mutations in axonal transport factors and cytoskeletal proteins can cause ALS and axonal transport defects have been observed in different ALS models (Alami *et al*, 2014; Baldwin *et al*, 2016; Guo *et al*, 2019; Sleigh *et al*, 2020). Using mitochondrial motility as an established readout for axonal transport, we found that mutant TDP‐43 motor neurons exhibit a decrease in mitochondrial transport. Correcting the pathogenic mutation in one of the mutant TDP‐43 lines rescued the observed changes in TDP‐43 biochemistry and the axonal transport deficit, suggesting that both are causally linked and are early manifestations of the disease‐causing gene mutation.

The fact that gene editing could revert both pathological and functional defects in our model indicates that we could use the iPSC‐derived motor neurons as a preclinical model to investigate the feasibility of therapies designed to halt the disease onset by interfering with the early steps in the pathological cascade. HDAC6 inhibition is one such promising therapeutic approach (Hubbert *et al*, 2002; Shen *et al*, 2016; Guo *et al*, 2017; Van Helleputte *et al*, 2018; Rossaert & Van Den Bosch, 2020). HDAC6 plays a role in aggresome formation and clearance of misfolded proteins (Pandey *et al*, 2007; Yan, 2014). Based on this role, inhibition of HDAC6 has previously been tested successfully in preclinical models of other neurodegenerative diseases related to protein aggregation, such as Alzheimer's disease, Parkinson's disease, hereditary spastic paraplegia, polyglutamine diseases, and Charcot‐Marie‐Tooth disease (Simões‐Pires *et al*, 2013; Guo *et al*, 2019). Indeed, treatment with Tubastatin A significantly reduced TDP‐43 mislocalization and the accumulation of insoluble TDP‐43 in mutant motor neurons. In addition, it rescued the mitochondrial transport defects observed in our TDP‐43 iPSC model, further supporting a link between TDP‐43 proteinopathy and axonal transport deficits. All these findings were mirrored by our TDP‐43 interactome analysis, where HDAC6 inhibition was able to normalize the mutant TDP‐43 interactome. However, the exact neuroprotective mechanisms of HDAC6 inhibition remain only partially understood. HDAC6 inhibition has also been reported to promote axonal transport, by increasing the acetylation of tubulin tracks through inhibition of its deacetylation by HDAC6 (D’Ydewalle *et al*, 2011; Prior *et al*, 2017; Guo *et al*, 2019). Additionally, HDAC6 has many other substrates and plays an important role as linker between ubiquitinated misfolded proteins and dynein motor proteins allowing for retrograde transport and degradation by autophagy (Yan, 2014). In summary, our data link axonal transport defects and TDP‐43 pathology in ALS and show that HDAC6 inhibition can counteract the early stages of TDP‐43 pathology. Therefore, we believe that HDAC6 inhibitors, when able to cross the brain–blood barrier, would be a promising therapeutic option for TDP‐43 proteinopathies.

## Materials and Methods

### Generation and characterization of TDP‐43 iPSCs

Three (G287S (P1), N290S (P2), and A382T + C9ORF72 (P3)) mutant TDP‐43 iPSC lines were generated in collaboration with the Stem cell institute, KU Leuven. Briefly, patient‐derived primary dermal fibroblasts were obtained through skin biopsy for which approval of the local ethical committee was obtained. These fibroblasts were genetically reprogrammed by Sendai virus‐mediated expression of embryonic stem cell‐specific genes as well as addition of embryonic stem cell defining factors (Klf4, Oct3/4, Sox2, and cMyc) using the CytoTune®‐iPS 2.0 Sendai Reprogramming Kit (A16517, Thermo Fisher Scientific, Waltham, Massachusetts, USA). Absence of Sendai virus after reprogramming was checked with quantitative real‐time polymerase chain reaction (qPCR) (Appendix Fig S7C). qPCR together with immunohistochemistry analysis was carried out to check for markers of pluripotency (e.g., Nanog, Oct4, SOX2, and SSEA4) (Appendix Fig S7D and E). In addition, the three germ layers were also evaluated by using the embryonic body formation assay, and also, the presence of self‐renewal capacity was observed (Appendix Fig S7F). Colonies with compact human ES cell (hESC)‐like morphology were expanded, and clonal lines were established for the three TDP‐43‐mutant lines. All lines underwent GCH array to exclude chromosomal alterations (Appendix Fig S8A–C). Additionally, we also performed repeat‐primed PCR to evaluate the repeat expansion in the mutant P3 iPSC line. The repeat‐primed PCR showed a pattern compatible with a long repeat expansion, with a repeat size of at least 60 units (Appendix Fig S8D).

### Differentiation iPSCs to motor neurons

Mutant and wild‐type control iPSCs were differentiated into motor neurons according to a well‐established protocol as described (Maury *et al*, 2015; Guo *et al*, 2017; Vandoorne *et al*, 2019; Fumagalli *et al*, 2019) (Appendix Fig S1A). The differentiation proceeds in three main steps: neuronal induction and caudalization (day 0–2), ventralization (day 2–9), and terminal differentiation and maturation (day 10 until day 38). Neural induction and caudalization were performed by adding neuronal basic medium (a 1:1 mixture of DMEM/F12 medium (31330038, Thermo Fisher Scientific) and Neurobasal medium (21103049, Thermo Fisher Scientific)), with N2 (17502‐048, Thermo Fisher Scientific) and B27 without vitamin A (12587‐010, Thermo Fisher Scientific), supplemented with 40 µM SB431542 (Tocris Bioscience, Bristol, UK), 0.2 µM LDN‐193189 (Stemgent, Cambridge, Massachusetts, USA), 3 µM CHIR99021 (Tocris Bioscience), and 5 µM Y‐27632 (Merck Millipore, Burlington, Massachusetts, USA). Ventralization of neuronal precursor cells was performed by adding 0.1 µM retinoic acid (Sigma) and 500 nM SAG (Merck Millipore). At day 10, the cells are replated onto laminin (20 μg/ml)‐coated 12‐well plates at a concentration of 0.5 × 10^5^ cells per well. Terminal motor neuron differentiation and maturation were performed by adding BDNF (500‐P84), GDNF (500‐P81), and CNTF (450‐13) (each 10 ng/ml, Peprotech, London, UK).

### Cell cultures

iPSCs were kept in Essential™ 8 medium (E8 flex) (A1517001, Thermo Fisher Scientific) supplemented with E8 supplement and penicillin–streptomycin (1%, Thermo Fisher Scientific). Cells were incubated at 37°C and 5% CO_2_. Splitting of cells, when confluent, was performed by a short wash with Dulbecco’s phosphate‐buffered saline (DPBS) followed by incubation of the cells with 0.5 mM EDTA (15575‐020, Thermo Fisher Scientific) in DPBS during 3 min at 37°C. Next, E8 flex medium was added and cells were detached using a cell scraper (734‐0385, VWR, Leuven, Belgium). The desired number of cells was transferred to a new Geltrex^R^ LDEV‐Free hESC‐Qualified Reduced Growth Factor Basement Membrane Matrix (A1413302, Thermo Fisher Scientific) coated well.

### Immunofluorescent staining

iPSC‐derived motor neurons were fixed at differentiation day 38. Briefly, cells were washed with DPBS and fixed with paraformaldehyde 4% (PFA) during 15 min at room temperature. Cells were then washed 3× with DPBS, followed by a blocking step with 5% normal donkey serum (D9663, Sigma‐Aldrich, Missouri, USA) and incubated with a primary antibody (Appendix Table S3: list of used antibodies and concentration details) at +4°C overnight. The next day the cells were washed 3× with DPBS, and a secondary antibody was added for 1 h and incubated at room temperature. As a negative control, one condition of fixed cells without a primary antibody but in the presence of a secondary antibody was imaged. In this way, we confirm the specificity of the primary antibody used. Before mounting the slides, the cells were treated with prolong gold (P36934, Thermo Fisher Scientific), for 20 min at room temperature, subsequently the cells were washed and mounted. The slides were imaged using an inverted confocal microscope (DMi8, Leica Microsystems, Mannheim, Germany) at room temperature. Confocal images of blue fluorescence were captured using 358 nm excitation light, images of red fluorescence were collected using 555 nm excitation light, and green fluorescence images were collected using 488 nm excitation light. As a negative control, one condition of fixed cells without a primary antibody but in present of a secondary antibody was imaged. Captured images were afterward analyzed using ImageJ software.

### TDP‐43 mislocalization on staining

The analysis was done in the DAPI channel (= unbiased method). The specific DAPI signal (=nucleus) was used to trace the nucleus. And if you use a filter on the DAPI channel (imageJ > fire filter) you also get a clear delineation of the cytoplasm (= non‐specific signal from DAPI). By doing everything on one channel and redirecting to the channel of interest, the measurement is therefore completely unbiased. In Addition, the value measured is the integrated density which is equal to product of the surface and the mean gray value.

### RNA sequencing

RNA sequencing was performed by the Nucleomics Core Facility (VIB, Leuven, Belgium) on iPSC‐derived motor neurons. RNA was isolated using an RNeasy kit (Qiagen). From extracted RNA, libraries were made using the Illumina TruSeq Stranded mRNA Library protocol. These libraries were sequenced on an Illumina NextSeq 500 paired‐end 75 bp and yield an average of 90 million reads per sample. To estimate the expression of the transcript of every sample, reads were counted using Salmon (v0.8.1) (Patro *et al*, 2017) against the ensemble transcript for the human reference genome hg38. Gene expression from the protein‐coding transcripts was then estimated using the tximport function the R‐package tximport (v1.6.0) (Soneson *et al*, 2016). Bulk RNA‐sequencing data of iPSCs and astrocytes were obtained from GSE98290 and processed in the same way (Hall *et al*, 2017).

### Live cell imaging of mitochondrial transport

To measure mitochondrial axonal transport, iPSC‐derived motor neurons at differentiation day 38 were washed with DPBS and incubated with 50 nM MitoTracker‐RED (M22425, Thermo Fisher Scientific) for 20 min at room temperature. Next, cells were incubated in motor neuron maturation medium. During imaging, the motor neurons were perfused in HEPES solution (pH 7.4, 150 mM NaCl, 5 mM KCl, 1 mM MgCl_2_, 2 mM CaCl_2_, 10 mM glucose, 10 mM HEPES) and a heated gravity‐fed perfusion system (Multichannel systems, Reutlingen, Germany) was used to maintain a temperature of 37°C. Images were taken using an inverted Zeiss Axiovert 200 M microscope (Carl Zeiss) with a 40× water immersion lens. The MitoTracker‐RED was excited at 580 nm, using a TILL Poly V light source (TILL Photonics), and image sequences were recorded (200 images at 1 Hz) with the help of a cooled CCD camera (PCO Sensicam‐QE) using TillVisION (TILL Photonics) software. Subsequently, mitochondrial movement along motor neuron axons was registered. The motor neurons were selected under differential interference optics (DIC) based on typical morphology consisting of a soma and long‐extended neurites.

Axonal transport video files were subsequently analyzed with Igor Pro (Wavemetrics) using custom‐written routines and a time/distance kymograph to quantify the number of stationary and moving mitochondria. Kymographs and spatiotemporal maps were created from the recorded videos, and subsequently, the number of moving and stationary mitochondria were subtracted. The moving mitochondria are tilted lines while the stationary mitochondria can be discerned as straight vertical lines.

### Subcellular fractionation

iPSC‐derived motor neurons were subcellularly fractionated into cytosolic and nuclear fractions. First, motor neurons were washed with ice‐cold DPBS and treated with 350 µl cytosolic extraction in 1 well of a 6‐well plate buffer (50 mM Tris–HCl, pH 6.5, 100 mM NaCl, 300 mM Sucrose, 3 mM MgCl_2_, 0.15% NP40, 4 mM DTT, 40 mM EDTA, and protease inhibitor) and transferred into a 1.5 ml Eppendorf. Then, those cells were rotated for 5 min at +4°C. After, rotation the cells were centrifuged at +4°C for 5 min at 5,000 *g*. The supernatant containing the cytosolic fraction was transferred into a new 1.5 ml Eppendorf and centrifuged at 10,000 *g* for 10 min at +4°C. While the pellet was kept to extract the nuclear proteins. Pellet was washed with cytosolic extraction buffer and rotated for 5 min at +4°C and centrifuged at 5,000 *g* for 5 min at +4°C. Remove supernatant as much as possible and add 250 µl of cytosolic extraction buffer to wash the pellet, centrifuge again at 5,000 *g* for 5 min at +4°C. Afterward, the supernatant was removed and 250 µl of the cytosolic extraction buffer was added to wash the pellet. After a centrifugation step of 5,000 *g* for 5 min at +4°C, the supernatant was removed completely, RIPA lysis buffer (50 mM Tris–HCl, pH 7.4, 100 mM NaCl, 1% Igepal CA‐630 (I8896, Sigma‐Aldrich), 0.1% SDS, 0.5% sodium deoxycholate, 4 mM DTT) was added, and the pellet was frozen as nuclear fraction.

### Western blot

Cells were washed with DPBS and suspended in RIPA buffer (R0278‐500ML, Sigma‐Aldrich) supplemented with protease inhibitor (cOmplete™, EDTA‐free protease inhibitor cocktail, 1836170001, Sigma‐Aldrich) and phosphatase inhibitor (PhosSTOP™, 4906837001, Sigma‐Aldrich). Protein quantification was performed using the Micro BCA™ Protein Assay Kit (23235, Thermo Fisher Scientific) according to the manufacturer’s instructions. Subsequently, samples were supplemented with SDS containing reducing sample buffer (39000, Thermo Fisher Scientific), denatured for 10 min at 95°C, and loaded on a 12% sodium dodecyl sulfate–polyacrylamide gel. After electrophoresis (100V, 1.5 h), the gel was transferred to a PVDF membrane (IPVH00010 Immobilon‐P transfer membrane, Sigma‐Aldrich) at 0.18 A during 1 h 45 min. Next, a blocking step was introduced with 5% nonfat dried milk (M7409‐1BTL, Sigma‐Aldrich) diluted in Tris‐buffered saline (T5912‐1L, Sigma‐Aldrich) with Tween (P1379‐500ML, Sigma‐Aldrich) (TBST) to reduce the background for 1 h at room temperature. The membrane was subsequently incubated with primary antibodies at +4°C overnight (except for the house keeping genes, where the membrane was incubated with primary antibodies for 1 h at room temperature) followed by washing 3 × 5 min in TBST. After washing step, a secondary antibody was applied for 1 h at room temperature. Subsequently, the membrane was washed 2 × 5 min with TBST and 1 × 5 min with TBS and treated with Pierce ECL (Peirce ECL, 32106, Thermo Fisher Scientific). The images were taken by using a chemiluminescence instrument (ImageQuant LAS400).

### CRISPR‐Cas9 gene editing: Isogenic controls and mCherry‐tagged cell lines

To correct the specific TDP‐43 heterozygous point mutations, we made use of the CRISPR‐Cas9 genome engineering technology as described before (Ordovás *et al*, 2015; Mancuso *et al*, 2019). Briefly, to accurately and efficiently target the gene of target we constructed a donor vector harboring the corrected point mutation and a selection cassette which we used to first select for positive clones with hygromycin (H3274‐50MG, Sigma‐Aldrich) as positive selector and afterward removing the cassette back from the positive clones with Fialuridine (FIAU) (SML0632‐5MG, Sigma‐Aldrich) as negative selector. The cassette together with 750 base pairs homology arms at both sides of the cassette was replaced in the target genome with homology recombination strategy. Afterward, excision of cassette was carried out by using piggyBac transposase technology which is known to result in a footprint‐free isogenic control cell line. PiggyBac transposase requires a TTAA sequence for its optimal function, and therefore, it is important to design the CRISPR‐Cas9 cutting site closed to TTAA sequence in the target genome. Next, we transfected 1 × 10^6^ iPSCs with donor plasmid (5 µg), gRNA plasmid (1 µg), and CRISPR‐Cas9 expressing plasmid (3 µg) using the Amaxa Nucleofector (Lonza, Basel, Switzerland), and the human embryonic stem cell Nucleofector Solution Kit 2 (VPH‐5022, Lonza) using the program F16. After 2–3 days post‐nucleofection, cells were selected with hygromycin for 7–10 days. Positive clones were genotyped with genomic DNA and used for further analysis of correct integration by sequencing. Removal of the cassette was done with piggyBac transposase. Therefore, cells from the positive clones were treated with 0.5 μM 1‐(2‐deoxy‐2‐fluoro‐beta‐D‐arabinofuranosyl)‐5‐iodouracil (FIAU) for 7 days and genomic DNA was collected for further analysis. At the end, the absence of the heterozygous TDP‐43 point mutation was confirmed and the positive clone was evaluated with CGH‐array (CME, UZ‐Leuven) to exclude chromosomal abnormalities induced by the genetic engineering. The same strategy was applied for creating MUT‐mCherry and WT‐mCherry‐tagged cell lines.

### Pull‐down mCherry‐tagged TDP‐43

For pull‐down of mCherry‐tagged TDP‐43, the RFP‐Trap Magnetic Agarose kit (rtma‐100, ChromoTek GmbH, Germany) was used according to the manufacturer’s instructions. Briefly, 25 µl of beads was first equilibrated by gently pipetting up and down and 500 µl ice‐cold dilution buffer (10 mM Tris/Cl pH 7.5, 150 mM NaCl, 0.5 mM EDTA). Afterward, the beads were separated with a Magnetic Separation Rack (A20006, NVIGEN Inc, San Jose, California, USA) until the supernatant was clear, the supernatant was discarded. iPSC‐derived motor neurons lysed in RIPA buffer supplemented with protease inhibitor (cOmplete™, EDTA‐free protease inhibitor cocktail, Sigma‐Aldrich) and phosphatase inhibitor (PhosSTOP™, Sigma‐Aldrich) were quantified using the Micro BCA™ Protein Assay Kit (23235, Thermo Fisher Scientific) according to the manufacturer’s instructions. In case of mCherry‐tagged pull‐downs, a commercially available kit was used (RFP‐Trap^®^ Magnetic Agarose Kit, rtmak‐20). The kit included its own negative control in which magnetic agarose beads are deactivated and they are made from the same magnetic agarose matrix as the RFP‐Trap magnetic agarose. Equal amount of diluted iPSC‐derived motor neuron lysate was added to the equilibrated beads and subsequently rotated overnight at +4°C. Next, day the beads were separated on the magnetic separation rack until the supernatant was clear, and beads were washed three times in 1 ml trypsin digestion buffer (20 mM Tris–HCl pH 8 and 0.2 mM CaCl_2_) before sent to the proteomics core.

### Sample preparation and LC‐MS/MS analysis

The samples were treated with 1 µg trypsin (V5111, Promega, Leiden, Netherlands), for 4 h at 37°C to cleave all proteins from the Ni beads. After removal of the Ni beads, the proteins were further digested with 1 µg trypsin overnight at 37°C. The resulting peptide mixture was acidified by addition of 1% trifluoroacetic acid (TFA) (Sigma‐Aldrich). Next, peptides were purified on OMIX C18 tips (A57003MB, Agilent, Santa Clara, USA). The tips were first washed 3 times with 150 µl pre‐wash buffer (0.1% TFA in water/ acetonitrile (ACN) (20:80, v/v)), and pre‐equilibrated five times with 150 µl of solvent A (0.1% TFA in water/ACN (98:2, v/v)) before samples were loaded on the tip. After peptide binding, the tip was washed three times with 150 µl of solvent A and peptides were eluted twice with 150 µl elution buffer (0.1% TFA in water/ACN (40:60, v/v)).

Purified peptides were re‐dissolved in 20 µl loading solvent (0.1% TFA in water/ACN (96:2, v/v)), and 5 µl of each sample was injected for LC‐MS/MS analysis on an Ultimate 3000 RSLCnano LC (Thermo Fisher Scientific) in line connected to a Q Exactive HF mass spectrometer (Thermo Fisher Scientific) equipped with a nanospray flex ion source (Thermo Fisher Scientific).

Trapping was performed at 10 μl/min for 4 min in loading solvent A on a 20 mm trapping column (Waters, nanoEase M/Z Symmetry C18 Trap Column, 180 μm internal diameter (I.D.), 5 μm beads). The peptides were separated on an in‐house produced column (75 µm × 500 mm) and packed in‐house with ReproSil‐Pur basic 1.9 µm silica particles (Dr. Maisch, Germany). The Ultimate 3000’s column oven was set to 50°C. For proper ionization, a fused silica PicoTip emitter (10 µm inner diameter) (New Objective) was connected to the analytical column. Peptides were eluted by a non‐linear gradient from 5 to 55% MS solvent B (0.1% FA in water/ACN (2:8, v/v)) over 87 min, at a constant flow rate 300 nl/min, followed by a 13‐minute washing phase plateauing at 99% MS solvent B. Re‐equilibration with 95% MS solvent A (0.1% FA in water) was performed at 300 nl/min for 20 min adding up to a total run length of 120 min. The mass spectrometer was operated in data‐dependent, positive ionization mode, automatically switching between MS and MS/MS acquisition for the 16 most abundant peaks in a given MS spectrum. The source voltage was 2.5 kV, and the capillary temperature was 275°C. One MS1 scan (m/z 375 − 1,500, AGC target 3 × 10^6^ ions, maximum ion injection time 60 ms), acquired at a resolution of 60,000 (at 200 m/z), was followed by up to 16 tandem MS scans (resolution 15,000 at 200 m/z) of the most intense ions fulfilling predefined selection criteria (AGC target 1 × 10^5^ ions, maximum ion injection time 80 ms, isolation window 1.5 Da, fixed first mass 145 m/z, spectrum data type: centroid, intensity threshold 1.3 × 10^4^, exclusion of unassigned, 1, 7, 8, > 8 positively charged precursors, peptide match preferred, exclude isotopes on, dynamic exclusion time 20 s). The HCD collision energy was set to 28% normalized collision energy, and the polydimethylcyclosiloxane background ion at 445.12003 Da was used for internal calibration (lock mass).

### Data analysis

Data analysis was performed with MaxQuant (version 1.6.11.0) using the Andromeda search engine with default search settings including a false discovery rate set at 1% on both the peptide and protein level (Cox & Mann, 2008; Cox *et al*, 2014). Spectra were searched against the human Swiss‐Prot database (from January 2020 with 20,365 entries). The mass tolerance for precursor and fragment ions was set to 4.5 and 20 ppm, respectively, during the main search. Enzyme specificity was set to C‐terminal of arginine and lysine, also allowing cleavage next to prolines with a maximum of two missed cleavages. Variable modifications were set to oxidation of methionine residues as wells as acetylation of protein N‐termini. Matching between runs was enabled with a matching time window of 0.7 min and an alignment time window of 20 min. Only proteins with at least one unique or razor peptide were retained. Proteins were quantified by the MaxLFQ algorithm integrated in the MaxQuant software. A minimum ratio count of two unique or razor peptides was required for quantification. Further data analysis was performed with the Perseus software (version 1.6.2.1) after uploading the protein groups file from MaxQuant (Tyanova *et al*, 2016). Reverse database hits were removed, and replicate samples were grouped. Proteins with less than three valid values in at least one group were removed, and missing values were imputed from a normal distribution around the detection limit. These quantified proteins were subsequently used for further data analysis.

### Gene Ontology enrichment analysis

Log2 fold changes were calculated between mCherry‐tagged wild‐type and mutant TDP‐43 interacting proteins and for mCherry‐tagged mutant TDP‐43 with or without Tubastatin A treatment. Log_2_ fold changes were converted to Z scores and ranked. A |Z‐score| > 1 was used as a cut‐off to select for enriched or depleted TDP‐43 interactors, and both lists were analyzed for gene ontology enriched terms using DAVID (Huang *et al*, 2009). Contaminants were removed from the gene enrichment analysis.

### Statistics

Comparisons between two groups were analyzed using paired ratio t‐test or unpaired Mann–Whitney test *P* < 0.05 were considered significant (*). Data are shown as mean, and error bars represent SEM of mean of a minimum three biological independent experiments. Statistical tests used are indicated in the figure legends. Results were plotted and analyzed using GraphPad Prism (v 7.01, GraphPad Software, Inc., San Diego, CA, USA).

## Author contributions

RF planned and performed most of the experiments and statistical analysis. SB helped with revising the manuscript and mass spectrometry analysis. AS revised the manuscript and performed the patch clamp measurements. MDD helped with TDP‐43 mislocalization experiments and analysis. LF helped with the iPSC differentiations and axonal transport experiments. MM helped with statistical analysis. JV helped with revising the manuscript. WG helped with iPSC differentiations. RB and TVe provided technical help with the tagged and isogenic control lines. KE helped with immunohistochemistry. BS revised the manuscript. JB helped with isogenic control and iPSC differentiations. DP revised the manuscript. TVa technical help with iPSC differentiation. PVB helped with axonal transport analysis software. CV provided ideas for the project. LVDB and PVD planned and supervised the project. RF and PVD wrote the first draft of the manuscript. All authors revised and approved the manuscript.

## Conflict of interest

The authors declare that they have no conflict of interest.

## Supporting information



Appendix

Source Data for Appendix

## Data Availability

The mass spectrometry proteomics data have been deposited to the ProteomeXchange Consortium via the PRIDE partner repository with the dataset identifier PXD023852 (http://www.ebi.ac.uk/pride/archive/projects/PXD023852). The transcriptome analysis can be found with dataset identifier EGAS00001003785 (https://ega‐archive.org/studies/EGAS00001003785), more specifically the gene expression of healthy control and mutant *TARDPB* iPSC‐derived motor neurons used in this manuscript under dataset identifier EGAD00001005216 (https://ega‐archive.org/datasets/EGAD00001005216).
