# Supplementary material for: HDAC6 inhibition restores TDP‐43 pathology and axonal transport defects in human motor neurons with TARDBP mutations
Source: EMBO J. 2021 Mar 10;40(7):e106177. doi: 10.15252/embj.2020106177 (PMC8013789; doi:10.15252/embj.2020106177)
Supplement: Supplementary file 1 — Appendix [file EMBJ-40-e106177-s001.pdf]

Appendix Figures and Tables

HDAC6 inhibition restores TDP-43 pathology and axonal transport defects in human motor neurons with *TARDBP* mutations

Raheem Fazal[1,2], Steven Boeynaems[3], Ann Swijsen[1,2], Mathias De Decker[1,2], Laura Fumagalli[1,2], Matthieu Moisse[1,2], Joni Vanneste[1,2], Wenting Guo[1,2,4], Ruben Boon[4], Thomas Vercruysse[5], Kristel Eggermont[1,2], Bart Swinnen[1,2,6], Jimmy Beckers[1,2], Donya Pakravan[1,2], Tijs Vandoorne[1,2], Pieter Vanden Berghe[7], Catherine Verfaillie[4], Ludo Van Den Bosch[1,2*] and Philip Van Damme[1,2,6*]

1. KU Leuven – University of Leuven, Department of Neurosciences, Experimental Neurology, and Leuven Brain Institute (LBI), Leuven, Belgium
2. VIB, Center for Brain & Disease Research, Laboratory of Neurobiology, Leuven, Belgium.
3. Department of Genetics, Stanford University School of Medicine, Stanford, California 94305, USA
4. Stem Cell Institute, Department of Development and Regeneration, Stem Cell Biology and Embryology, KU Leuven, Leuven, Belgium
5. KU Leuven Department of Microbiology, Immunology and Transplantation, Laboratory of Virology and Chemotherapy, Rega Institute for Medical Research, Leuven, Belgium
6. University Hospitals Leuven, Department of Neurology, Leuven, Belgium
7. KU Leuven, Department of Chronic Diseases, Metabolism and Ageing, Translational Research in GastroIntestinal Disorders, KU Leuven, Leuven, Belgium

KEYWORDS

TDP-43, induced pluripotent stem cells, wild-type and mutant tagged TDP-43, axonal transport, HDAC6.

Table of Contents

Appendix Figure S1……………………………………………………………………..3

Appendix Figure S2……………………………………………………………………..4

Appendix Figure S3……………………………………………………………………..5

Appendix Figure S4……………………………………………………………………..6

Appendix Figure S5……………………………………………………………………..7

Appendix Figure S6……………………………………………………………………..8

Appendix Figure S7……………………………………………………………………..9

Appendix Figure S8………………………………………………………………………10

Appendix Table S1……………………………………………………………………..11

Appendix Table S2……………………………………………………………………..11

Appendix Table S3……………………………………………………………………..12

Appendix Table S4……………………………………………………………………..13

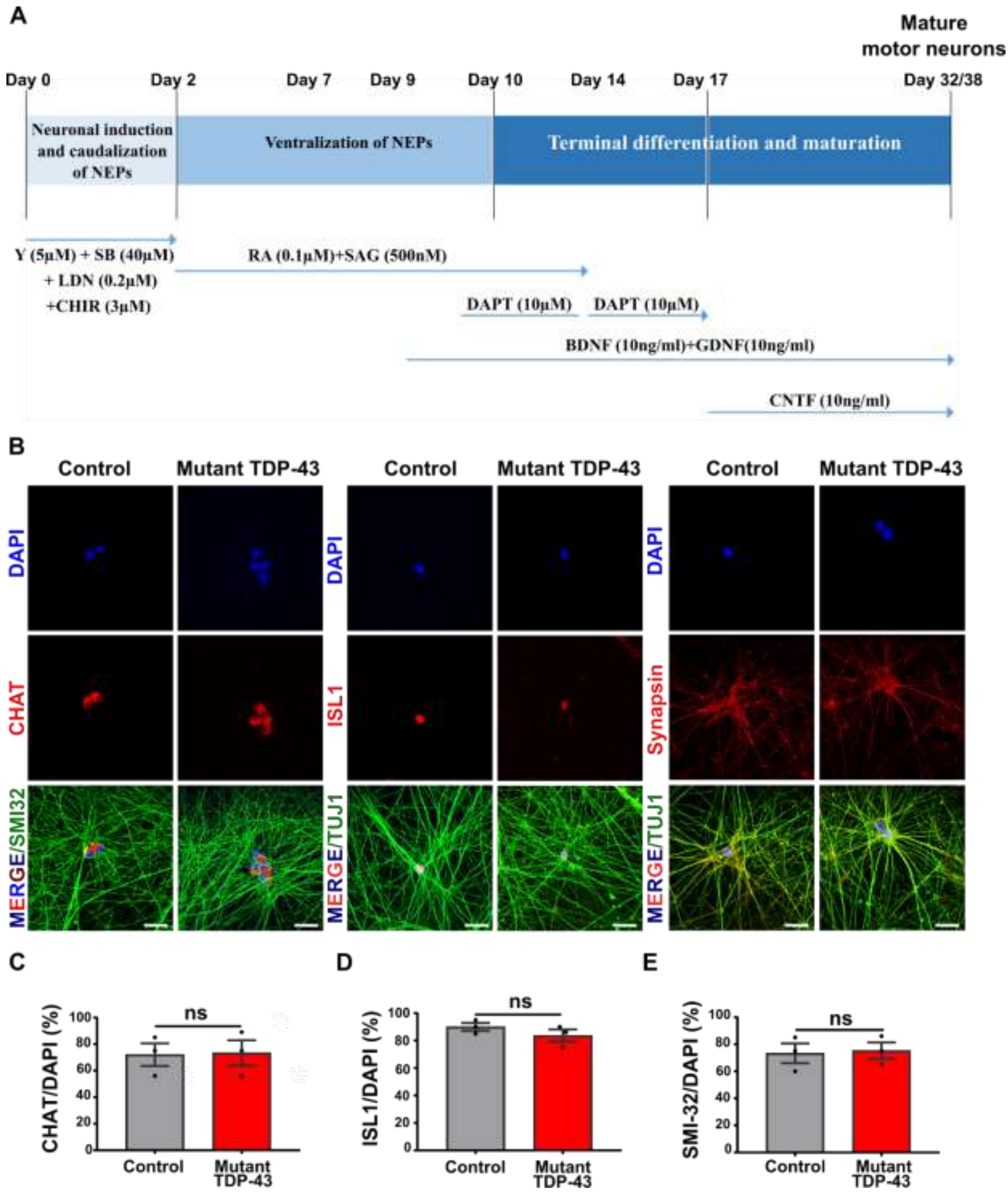

Appendix Figure S1. Mutant TDP-43 iPSCs differentiation into functional motor neurons. A Scheme representing the used protocol to differentiate iPSC to motor neurons. **B** Representative immunofluorescent staining of healthy control (C0) and mutant TDP-43 (P2) iPSC-derived motor neurons. Scale bar: 100µm. **C** Quantification of ISL1 positive cells, **D** quantification of CHAT positive cells and **E** quantification of SMI32 positive cells. Each dot represent the average of one differentiation with n=30 control (C0=10, C1=10 and C2=10) and n=30 mutant TDP-43 (P1=10, P2=10 and P3=10) cells counted per differentiation. Paired-t-test in all panels: Data was combined from three independent differentiations. Data are shown as mean ± SEM, ns: not significant.

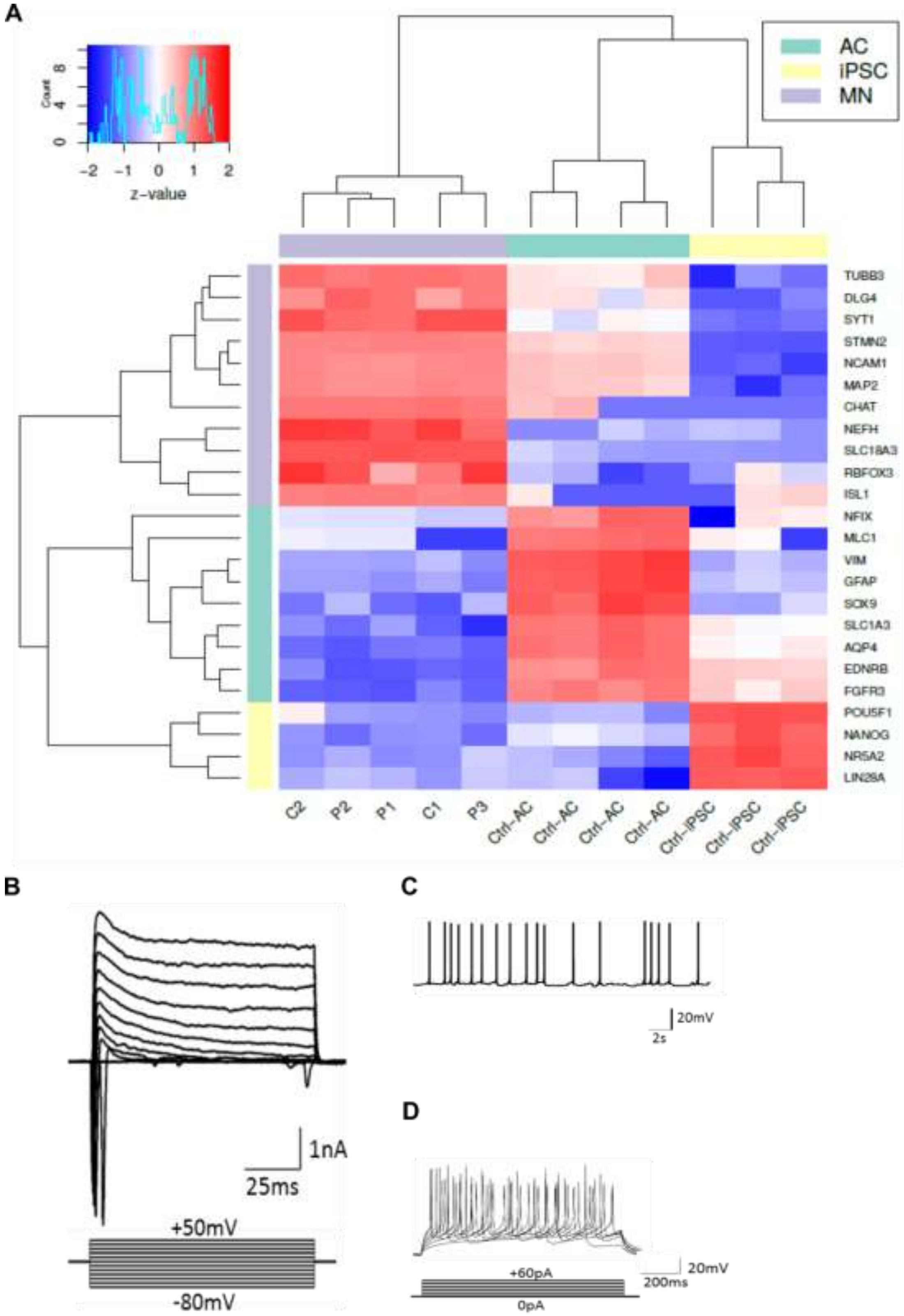

Appendix Figure S2. Mutant iPSC-derived motor neuron characterization. A RNA sequencing analysis in iPSC-derived motor neurons indicates exclusive enrichment of motor neuron specific transcripts versus iPSC -and astrocyte specific transcripts. **B** Representative traces of in- (Na^{+}) and outward (K^{+}) currents (top panel) elicited by an experimental voltage pulse step protocol (lower panel). **C** Representative trace of spontaneous action potentials. **D** Representative traces of action potentials (top panel) evoked by an experimental current pulse step protocol (lower panel).

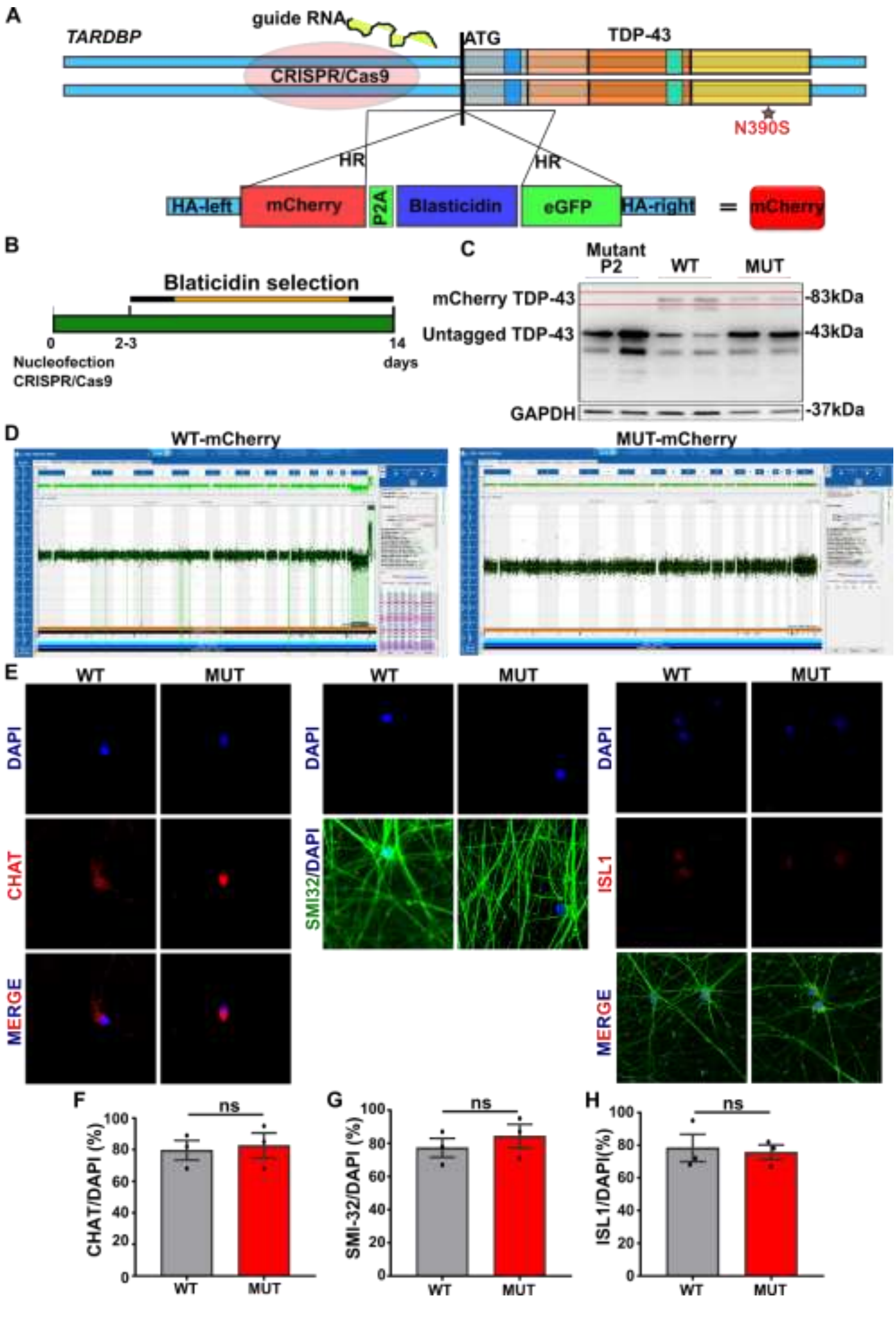

Appendix Figure S3. Generation of mCherry tagged mutant TDP-43 patient cell lines using CRISPR-Cas9 genome technology. A Overview of the used strategy to generate tagged lines with CRISPR/Cas9 genome editing. Donor template DNA with the selection cassette containing blasticidin and a highly similar sequence (homology arm-right and left), necessary for very efficient homology recombination with the target gene sequence. **B** Experimental design of blasticidin selection to select positive colonies. **C** Western blot confirming the expression of both the mCherry tagged TDP-43 as well as the untagged TDP-43 in the newly generated cell lines. **D** CGH array of WT-mCherry (left panel) and MUT-mCherry (right panel). **E** Immunohistochemistry to evaluate motor neuron specific markers. **F** Quantification of CHAT+, **G** quantification SMI-32+ and **H** quantification of ISL1+ cells. Each dot represents an independent differentiation. Ratio paired-t-test. For all panels: Data combined from three independent differentiations. Data are shown as mean ± SEM. ns: not significant.

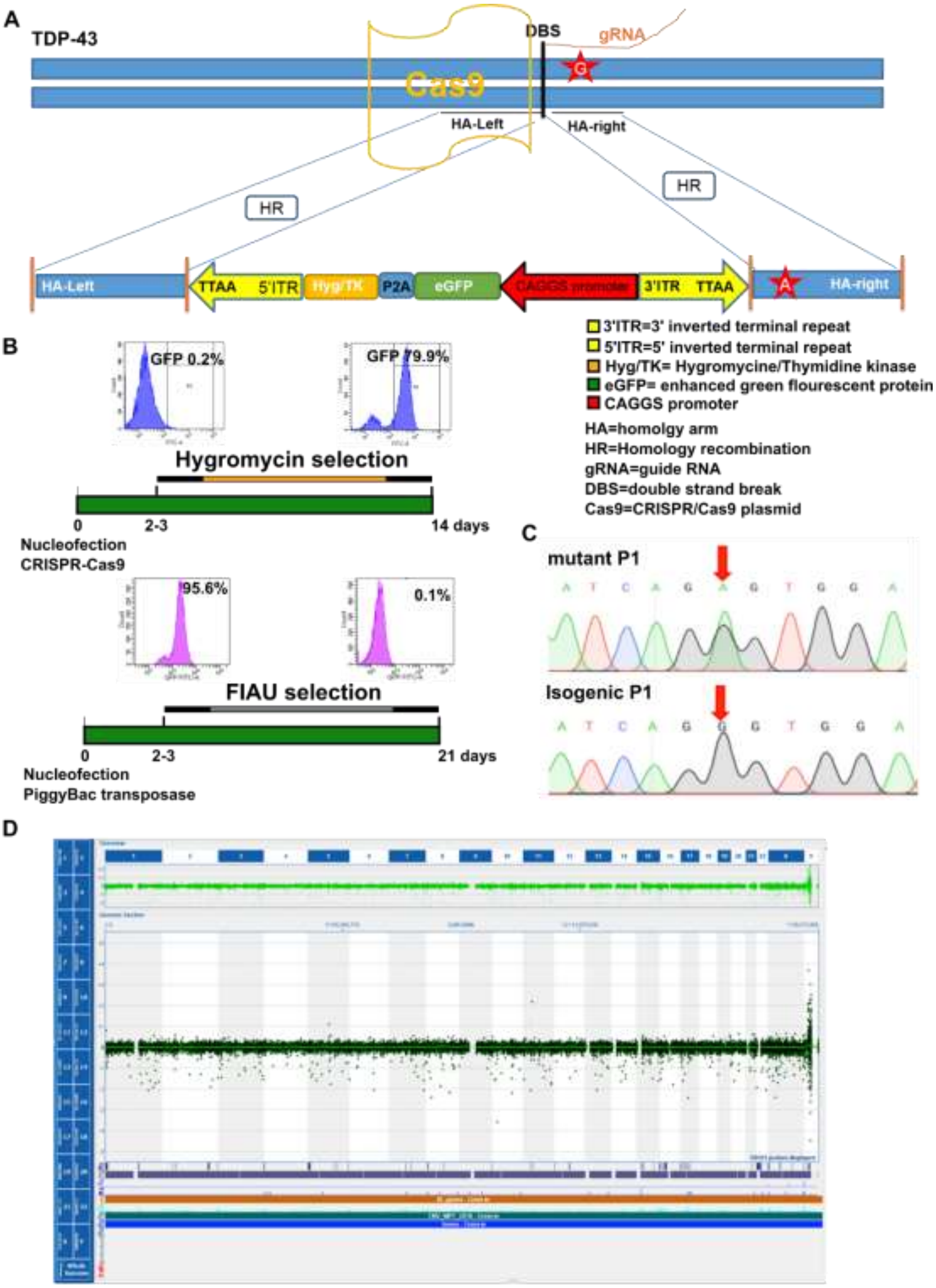

Appendix Figure S4. Generation of an isogenic, gene-corrected control line. A General overview of the experimental design and strategy used to generate isogenic control by correcting heterozygous point mutation with CRISPR/Cas9 genome editing. Donor template DNA with the selection cassette containing, hygromycin (positive selector), FIAU (negative selector) and containing highly similar sequence (homology arm-right and left), necessary for very efficient homology recombination with the target gene sequence. **B** Experimental design of hygromycin selection and piggyBac excision of the cassette from the positive clone, confirmed by fluorescence-activated cell sorting (FACS). **C** Confirming the presence of mutation by Sanger sequencing in mutant P1 (G287S) versus isogenic P1 (G287G) iPSC line. **D** CGH-array, indicating no significant genome-wide aberrations between mutant P1 and isogenic P1.

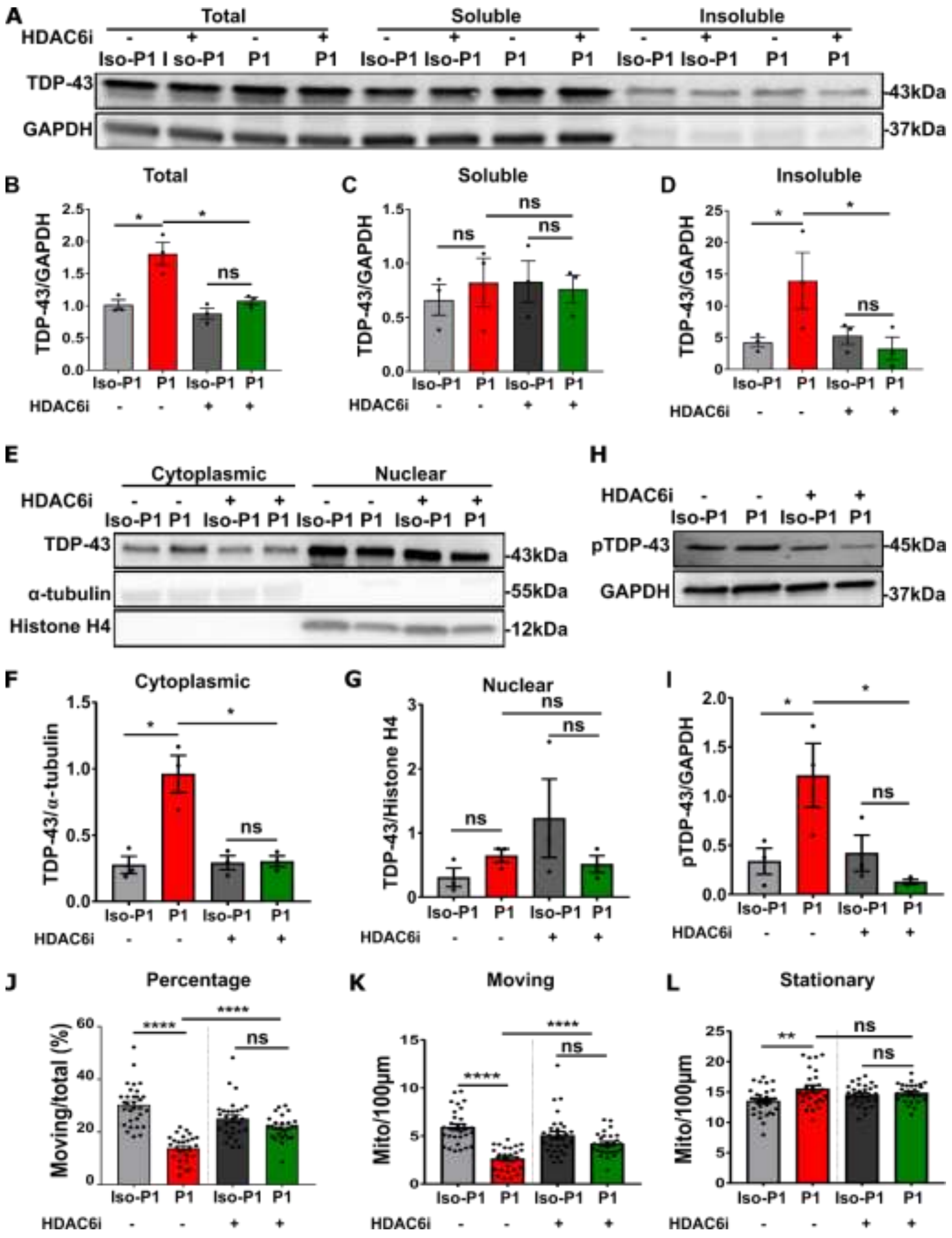

Appendix Figure S5. HDAC6 inhibitor rescues the observed TDP-43 pathology and axonal transport defects in the mutant TDP-43. A, **B**, **C** and **D** Representative Western blot of total, soluble and insoluble TDP-43 levels. (**B)** Quantification of total full-length TDP-43, (**C**) quantification of soluble full-length TDP-43, ratio paired-t-test and (**D)** quantification of insoluble full-length TDP-43 levels, ratio paired-t-test. Each dot represents an independent differentiation. **E** Representative Western blot of nucleo-cytoplasmic fractionation of mutant P1 and Iso-P1. **F** Graph shows quantification of cytoplasmic fraction. **G** Graph shows quantification of nuclear fraction, ratio paired-t-test. **H** Representative Western blot of pTDP-43 levels. **I** Graph shows quantification of pTDP-43, ratio paired-t-test. **J, K, L** Quantification of percentage of moving mitochondria (**J**), the absolute amount of moving mitochondria (**K**) and of the amount of stationary mitochondria (**L**) normalized to neurite length 100µm. Each dot represents one neurite with for Iso-P1 (n=31) versus mutant P1 (n=27) and Iso-P1 with HDAC6 inhibitor (n=29) versus mutant P1 with HDAC6 inhibitor (n=27), unpaired Mann-Whitney test. Data was combined from three independent differentiations. Data are shown as mean ± SEM, *p<0.05, **p<0.01, ****p<0.0001, ns: not significant.

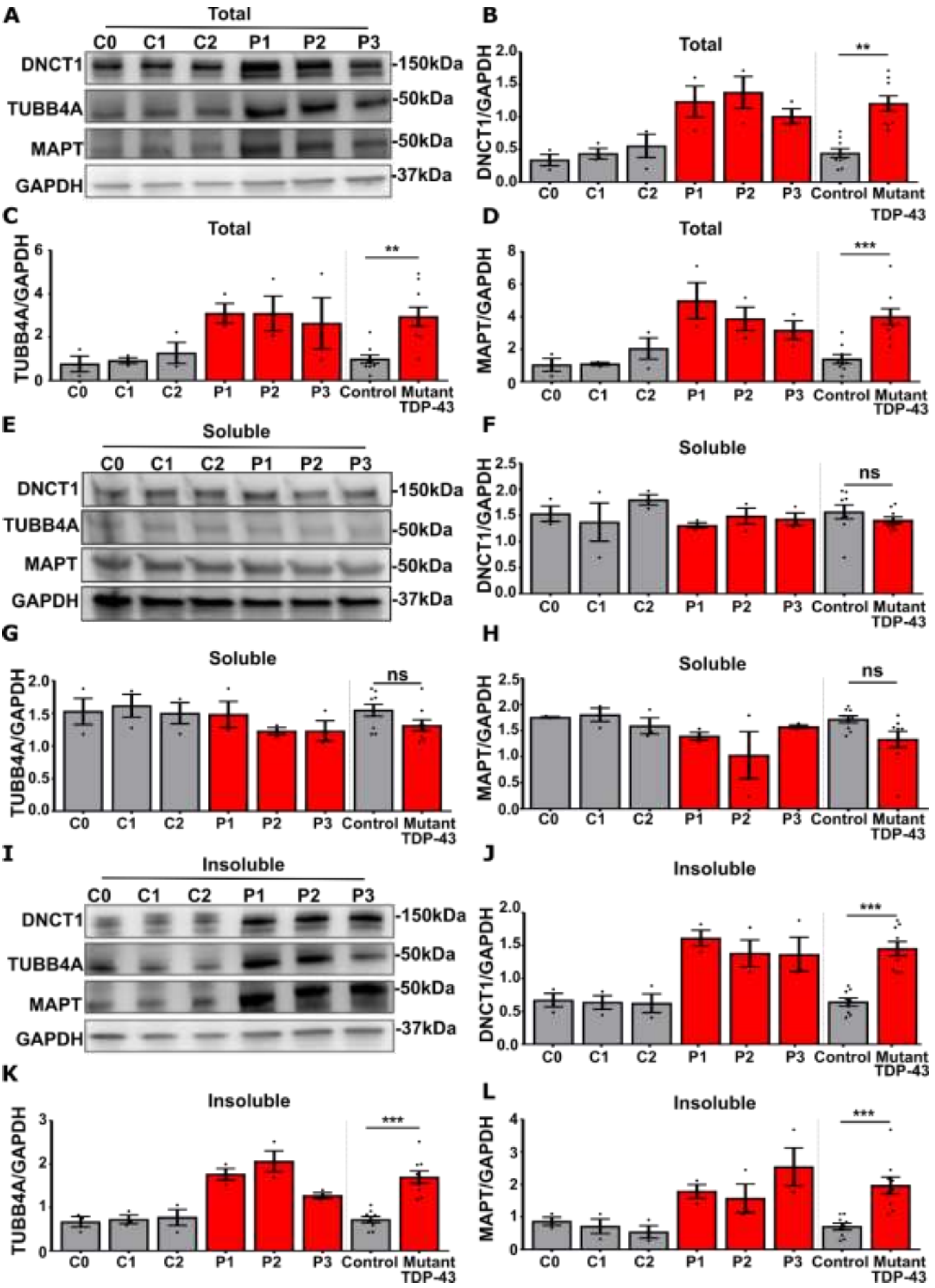

Appendix Figure S6. Validation of the interactome data. We studied the binding partners that favoured mutant TDP-43 in **A** total, **E** soluble and **I** insoluble fractions using Western blot of motor neurons derived from control and mutant TDP-43 iPSC lines. **B** quantification of dynactin 1 (DNCT1), **C** quantification of TUBB4A and **D** quantification MAPT in total fraction. **F** Quantification of DNCT1, **G** quantification of TUBB4A and **H** quantification MAPT in the soluble fraction. **J** Quantification DNCT1, **K** quantification of TUBB4A and **L** quantification of MAPT in the insoluble fraction. Each dot represents an independent differentiation. Ratio paired-t-test. For all panels: Data combined from three independent differentiations. Data are shown as mean ± SEM, $^{**}p<0.01$, $^{***}p<0.001$.

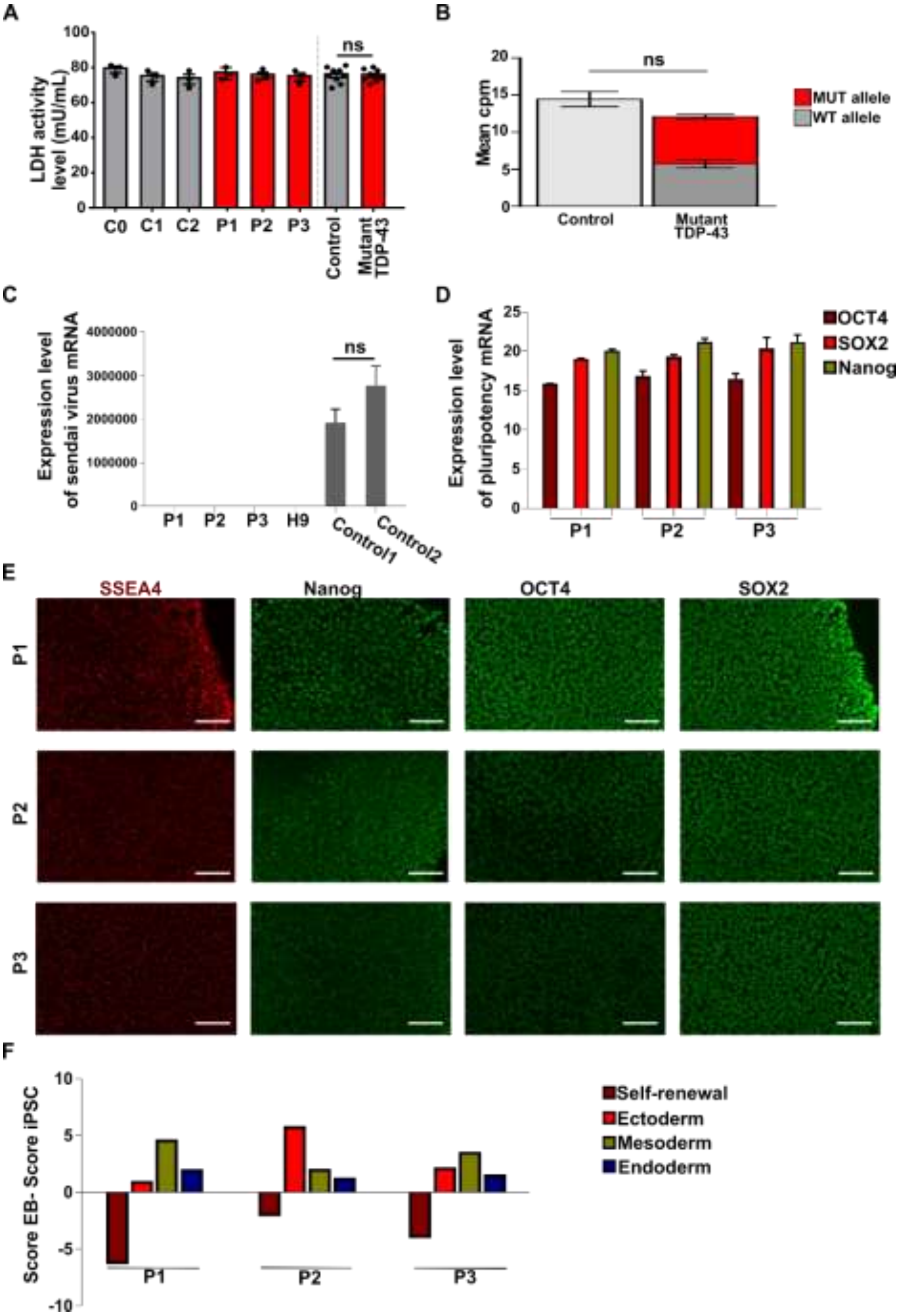

Appendix Figure S7. LDH assay, RNA expression of motor neurons and quality control of the generated iPSC lines from patient skin fibroblasts. A LDH assay, to compare cytotoxicity in control and mutant TDP-43 iPSC-derived motor neurons. Data from three independent differentiation, paired-t-test. Data are shown as mean ± SEM, ns: not significant. **B** Evaluation of TDP-43 RNA expression in the transcriptomic data between control (C1 and C2) and mutant TDP-43 (P1, P2 and P3) iPSC-derived motor neurons, data from one individual differentiation. Mutant TDP-43 bar is divided in two red and grey, representing mutant and wild type allele respectively, chi-squared test. Data are shown as mean ± SEM, ns: not significant. **C** Sendai virus PCR of the three mutant TDP-43 lines, a negative control (H9) and two positive controls (control1 and control2), unpaired Mann-Whitney test. Data from three different clones and shown as mean ± SEM. **D** Pluripotency PCR, data from different clones and **E** represented immunofluorescence staining of iPSC pluripotency markers, *i.e.* Nanog, SOX2, OTC4 and SSEA4. Scale bar: 25μm. **F** qPCR of embryonic body formation experiment showing the presence of the three germ layer markers, data collected from one clone of each iPSC line.

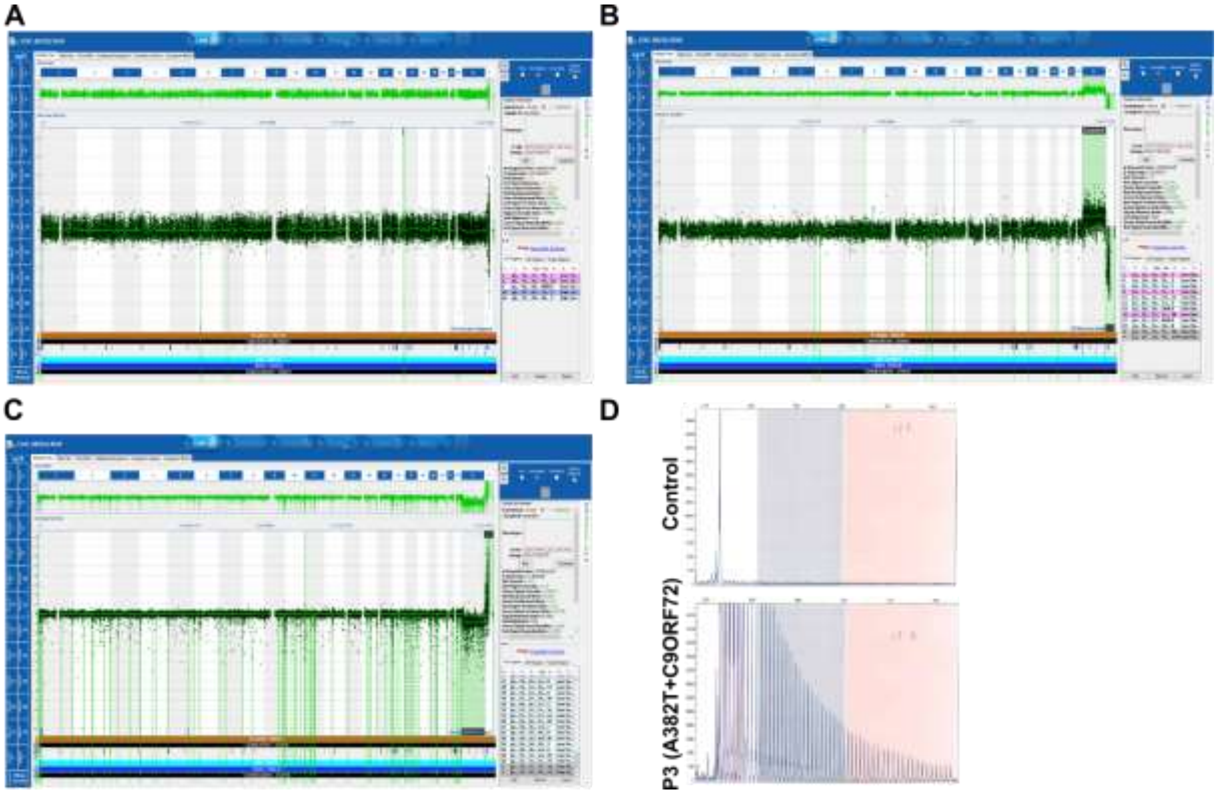

Appendix Figure S8. CGH array of the mutant TDP-43 and tagged iPSC lines. A (P1), **B** (P2) and **C** (P3). **D** Electropherograms of the PCR products of repeat-primed PCR reactions with control (upper-panel) and mutant P3 (lower-panel), evaluating the $GGCCCC_n$ repeat expansion.

Appendix Table S1. Overview of human iPSC lines used in this study.

Code	ALS mutation	Diagnosis	Gender	iPSC lines
C0	None	/	F	1
C1	None	/	F	1
C2	None	/	M	1
P1	G287S	TARDBP-ALS	F	2
P2	N390S	TARDBP-ALS	M	3
P3	A382T+C9ORF72 (>60 repeats)	TARDBP-ALS	F	3
Isogenic P1	G287G	TARDBP-ALS	F	1
WT-mCherry (P2)	N390S	TARDBP-ALS	M	1
MUT-mCherry (P2)	N390S	TARDBP-ALS	M	1

Appendix Table S2. List of depleted and enriched genes in MUT- versus WT-Cherry iPSC-derived motor neurons

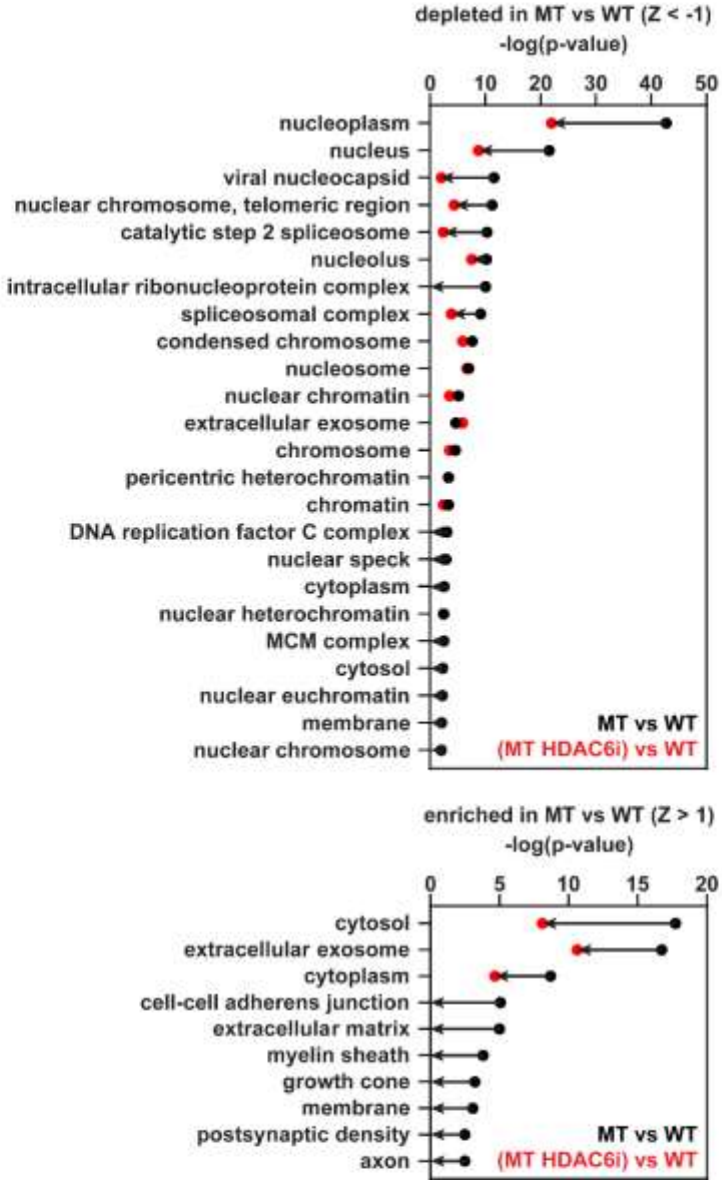

Appendix Table S3. List of antibodies used for (1) immunohistochemistry and (2) Western blot.

Antibody	Isotype	Dilution (1)	Dilution (2)	Source
Synapsin 1	Rabbit IgG	1/2000		Merck Millipore
Tuj1	Mouse IgG	1/500		Abcam
CHAT	Rabbit IgG	1/500	1/200	Merck Millipore
ISL1	Rabbit IgG	1/200	1/100	Merck Millipore
SMI32	Rabbit IgG	1/1000	1/500	Abcam
TARDBP	Rabbit IgG	1/200	1/500	Proteintech
pTARDBP	Mouse IgG	1/200	1/500	Proteintech
α-tubulin	Mouse IgG		1/5000	Abcam
Histone H4	Rabbit IgG		1/500	Abcam
GAPDH	Mouse IgG		1/5000	Abcam
Acetylated α-tubulin	Mouse IgG		1/5000	Abcam
SSEA4	Mouse IgG	1/200		Santa Cruz
Tra1-60	Mouse IgG	1/1000		Merck Millipore
OCT4	Rabbit IgG	1/400		Santa Cruz
Nanog	Goat IgG	1/500		R&D
SOX2	Goat IgG	1/500		R&D
DNCT1	Goat IgG		1/500	Abcam
TUBB4A	Rabbit IgG		1/1000	Mybiosource
MAPT	Rabbit IgG		1/250	lsbio

Appendix Table S4. Primers used in making of tagged lines, isogenic control, sequencing, Semi-quantify PCR and qPCR

Name	Forward primer sequence 5'---3'	Reverse primer sequence 5'---3'
Isogenic HA-left donor template	CTGCAGAAGGTGTAGACGTTGAGAG C	ATGCGTCATTTTGACTCACGCGGT
Isogenic HA-right donor template	TATTGACGTCAATGGGCGGGGG	CACAACCAGGCAACTACTCTCCCAG
Isogenic guide RNA TDP-43	CACCGAACTGCTCTGTAGTGCTGCC	AAACGGCAGCACTACAGAGCAGTT C
Isogenic TDP-43 amplification	GGGGTTTAAATGAAATGAGTGTTC	AAACAAAAGAACCAAACACTGTGA
Isogenic TDP-43 amplification nested PCR	GCCGAACCTAAGCACAATAGC	GAACCAAACACTGTGACACCA
Isogenic TDP-43 amplification nested PCR Sequencing	GAAGATTTGGTGGTAATCCAGG	CCAATCAGGCAAACAGCAG
Tagged lines HA-left Donor template	GATCTGGCTGGTCTTGAACTCC	CCTAAATGCACAGCGACGGA
Tagged lines HA-right Donor template	GGCGGGCCATTTACCGTAAG	CCTGTGATGCGTGATGACGA
Tagged lines cDNA amplification	AAGGGCGAGATCAAGCAGAGG	CCAATCAGGCAAACAGCAGTTCA
Tagged lines cDNA amplification sequencing	TCCCACAACGAGGACTACACC	CTATACCAACCAACCACAACCC
Tagged lines guide RNA	CACCGCCGAAGATGAGAACGATGA GC	AAACGCTCATCGTTCTCATCTTCGG C
OCT4	GATGGCGTACTGTGGGCCC	TGGGACTCCTCCGGGTTTTG
Nanog	CAGCCCCGATTCTTCCAGTCCC	CGGAAGATTCCCAGTCGGGTTCAC C
SOX2	GGGAAATGGGAGGGGTGCAAAAG AGG	GGGAAATGGGAGGGGTGCAAAAG AGG
Sendai	TGCCCCAAGCAGACACCACCTG GCA	
