# Supplementary figures and images for: HDAC6 inhibition restores TDP‐43 pathology and axonal transport defects in human motor neurons with TARDBP mutations
Source: EMBO J. 2021 Mar 10;40(7):e106177. doi: 10.15252/embj.2020106177 (PMC8013789; doi:10.15252/embj.2020106177)

Figure 1

Panel B

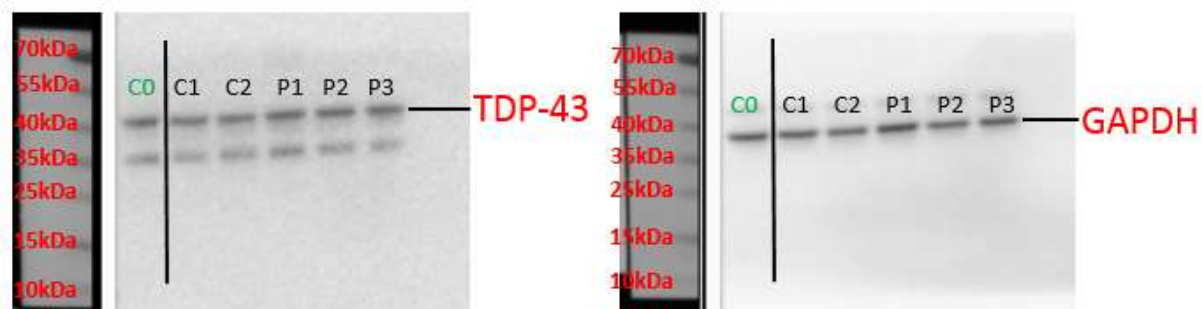

Panel D

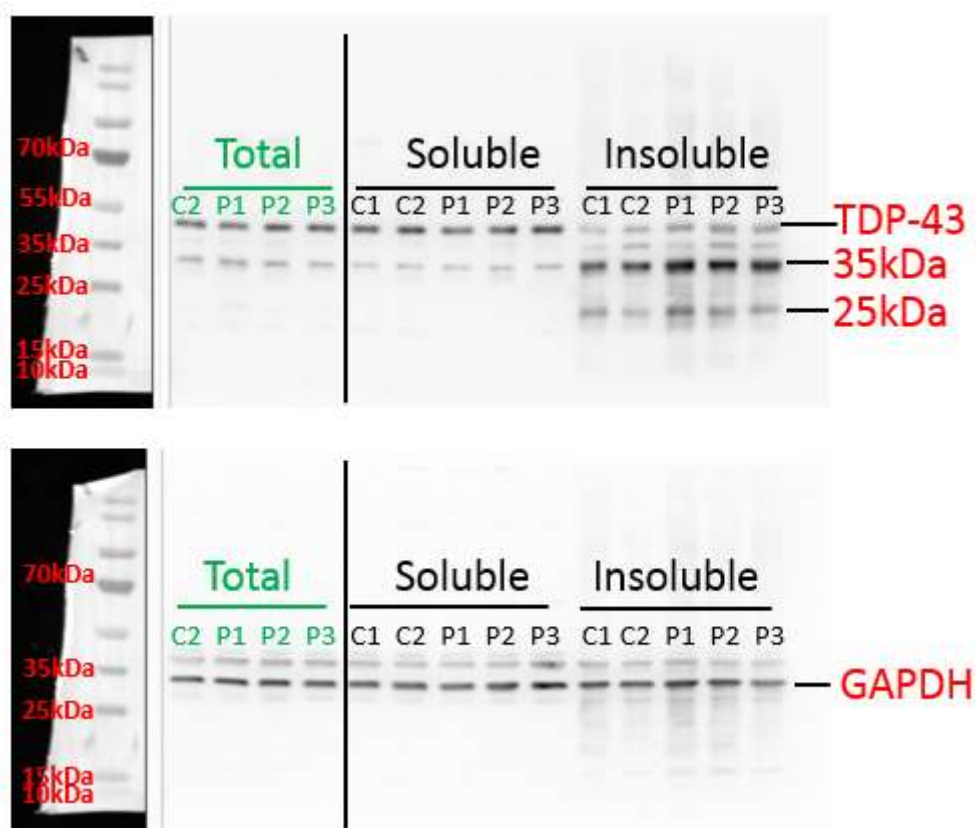

Supplement: Supplementary file 2 — Source Data for Appendix [file EMBJ-40-e106177-s002.zip › embj2020106177-sup-0002-SDataEV/embj2020106177-sup-0001-SDataFigS1.pdf]

Figure 2

Panel D

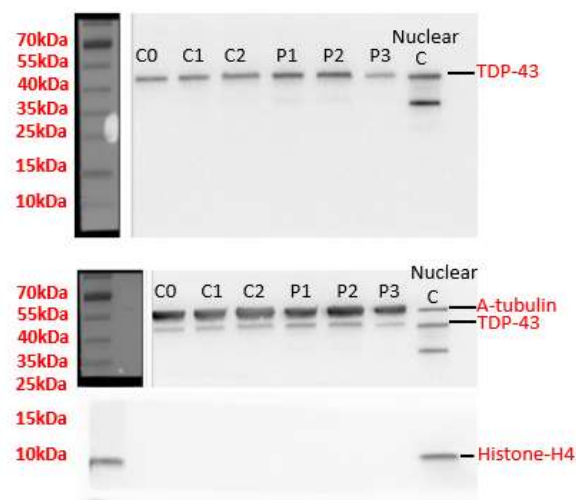

Panel F

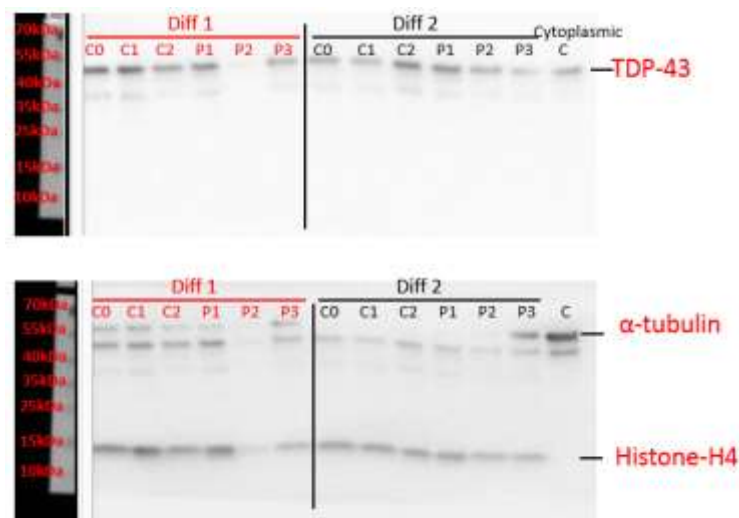

Panel K

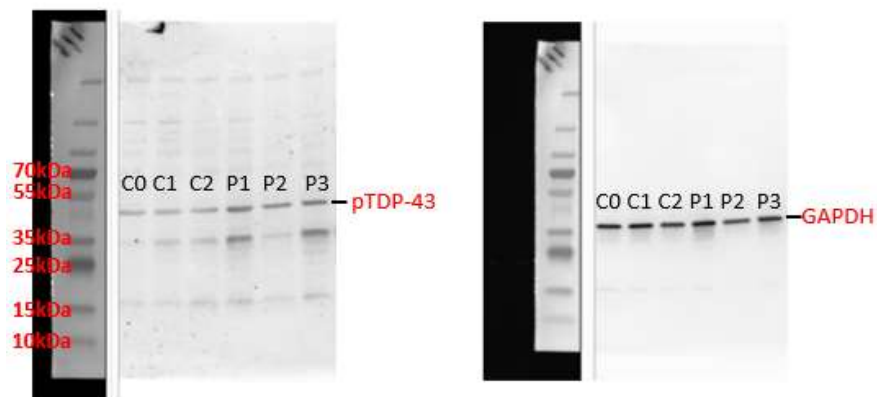

Supplement: Supplementary file 2 — Source Data for Appendix [file EMBJ-40-e106177-s002.zip › embj2020106177-sup-0002-SDataEV/embj2020106177-sup-0002-SDataFigS2.pdf]

Figure 3

Panel E

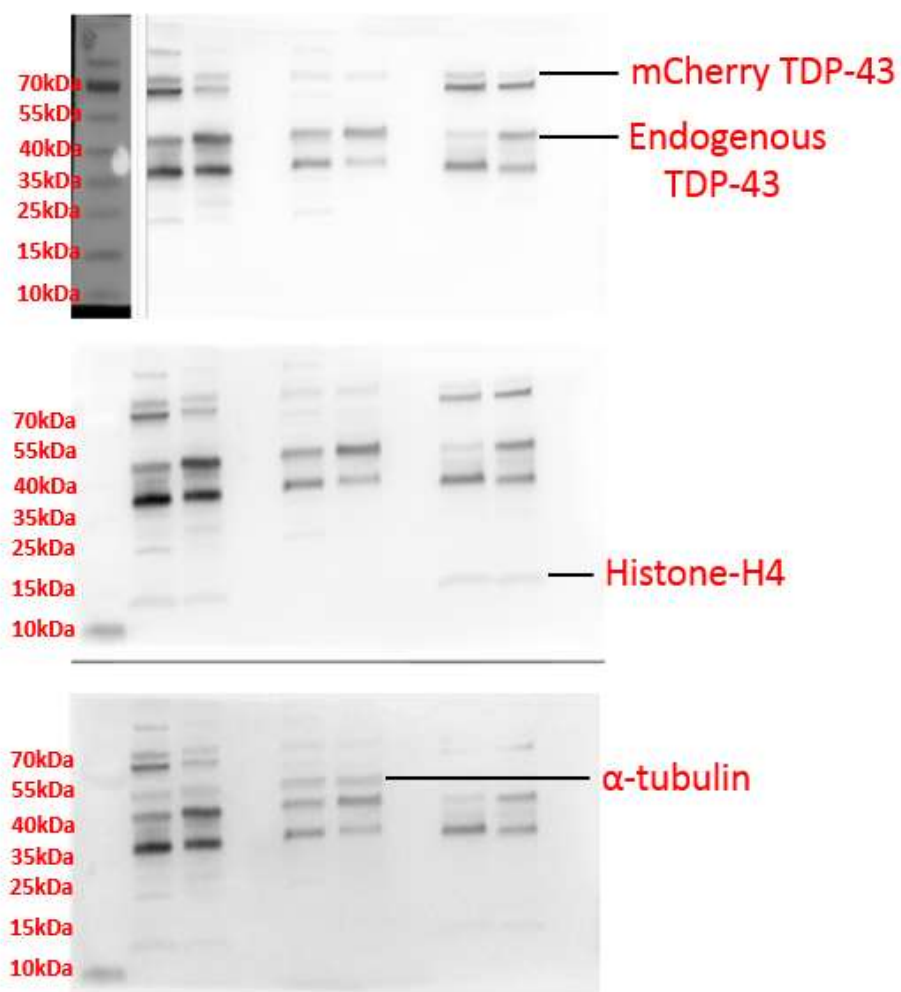

Panel H

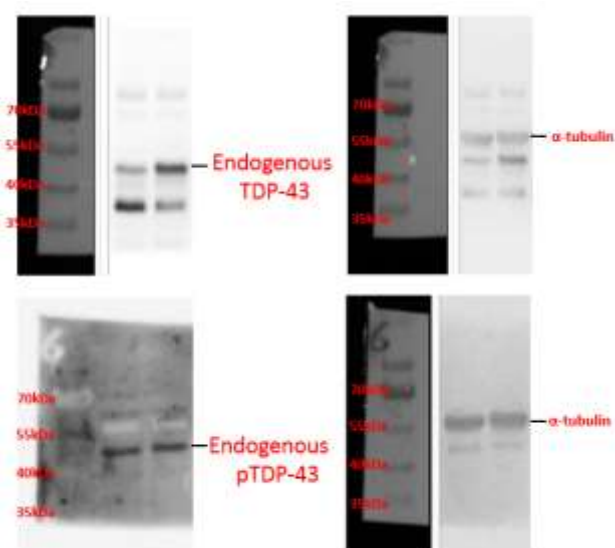

Supplement: Supplementary file 2 — Source Data for Appendix [file EMBJ-40-e106177-s002.zip › embj2020106177-sup-0002-SDataEV/embj2020106177-sup-0003-SDataFigS3.pdf]

Figure 5

Panel A

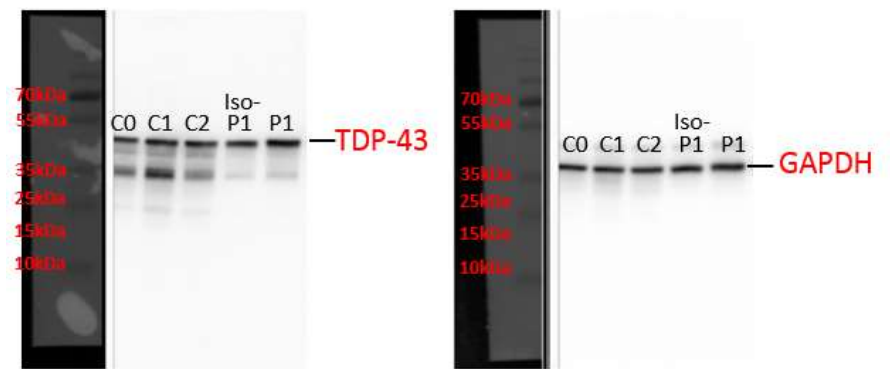

Panel C

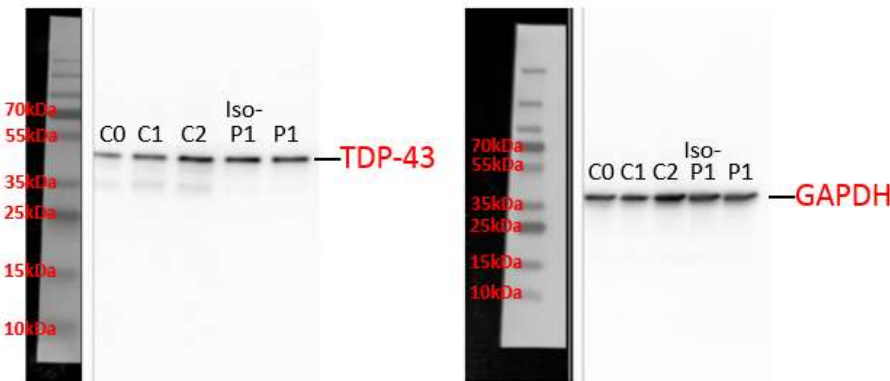

Panel E

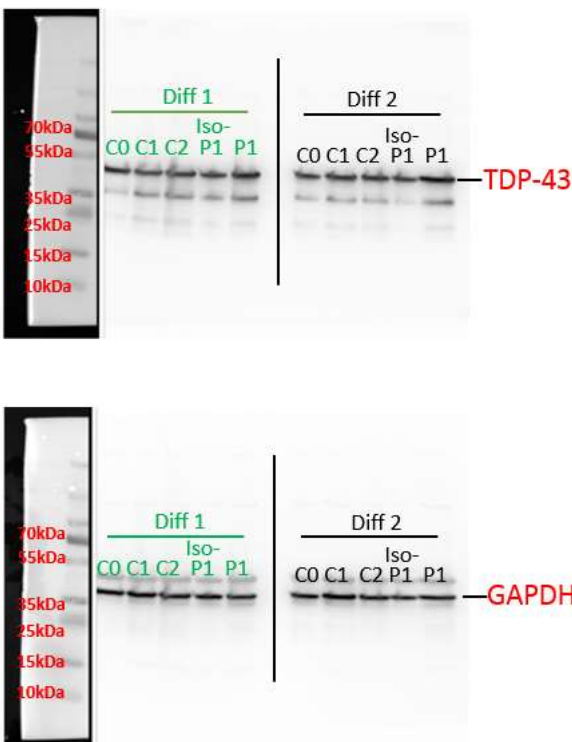

Supplement: Supplementary file 2 — Source Data for Appendix [file EMBJ-40-e106177-s002.zip › embj2020106177-sup-0002-SDataEV/embj2020106177-sup-0005-SDataFigS5.pdf]

Figure 6

Panel A

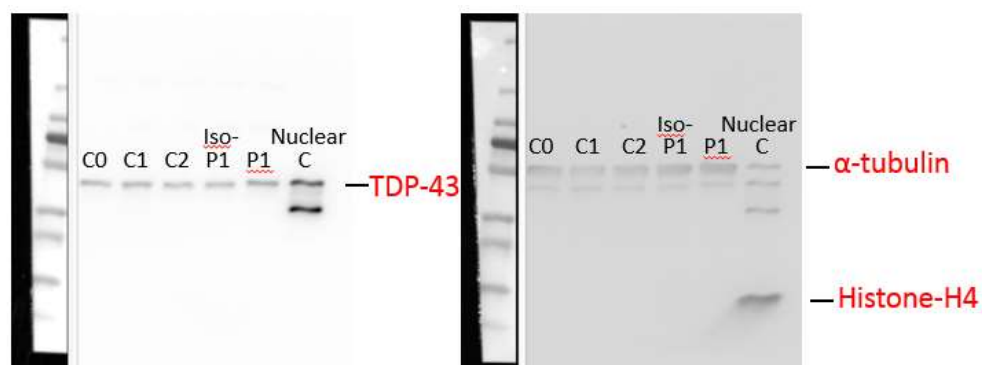

Panel C

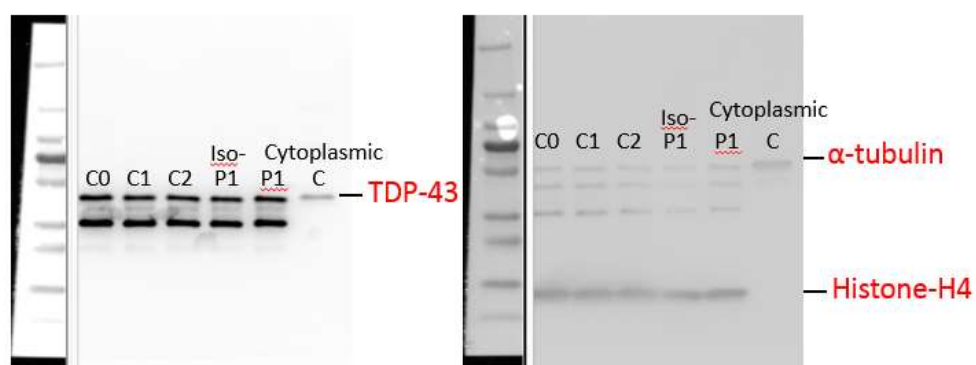

Panel H

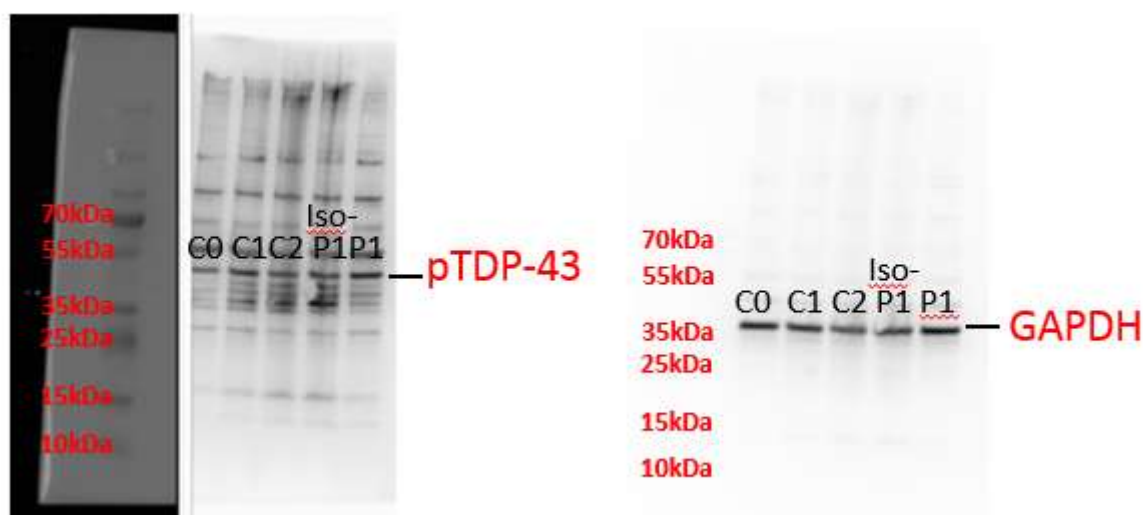

Supplement: Supplementary file 2 — Source Data for Appendix [file EMBJ-40-e106177-s002.zip › embj2020106177-sup-0002-SDataEV/embj2020106177-sup-0006-SDataFigS6.pdf]

Figure 7

Panel A

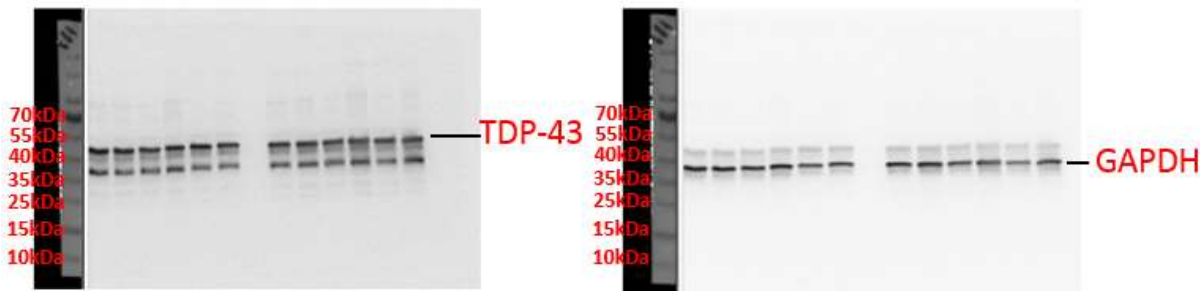

Panel C

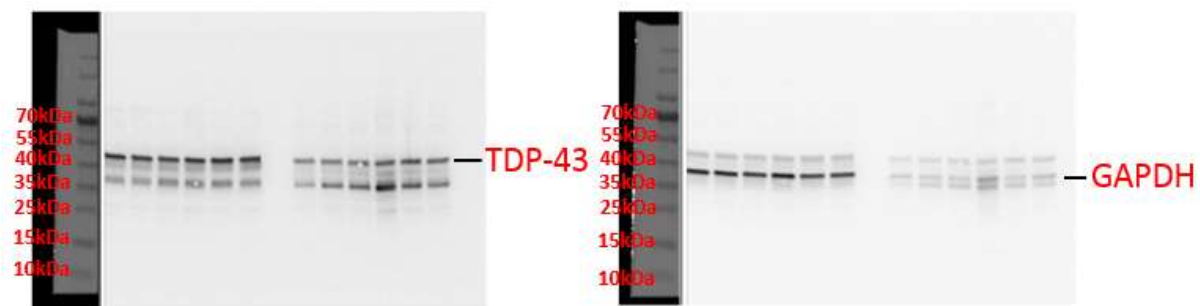

Panel E

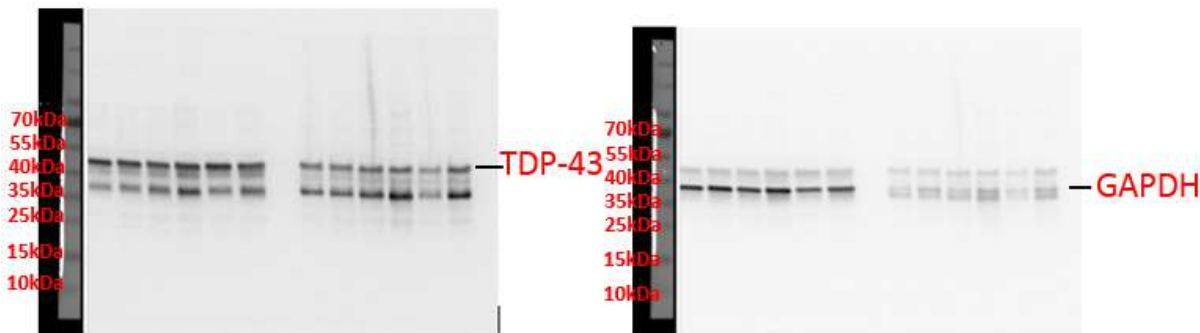

Panel G

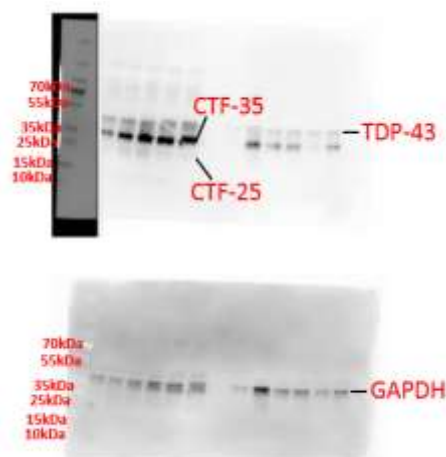

Supplement: Supplementary file 2 — Source Data for Appendix [file EMBJ-40-e106177-s002.zip › embj2020106177-sup-0002-SDataEV/embj2020106177-sup-0007-SDataFigS7.pdf]

Figure 8

Panel D

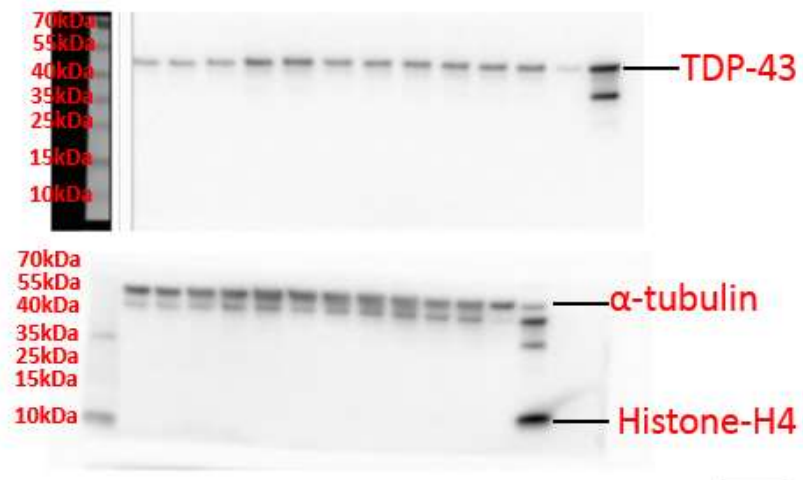

Panel F

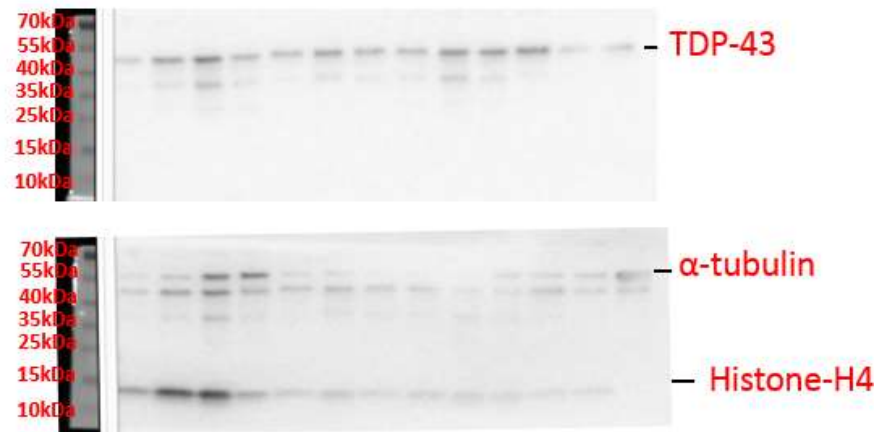

Panel H

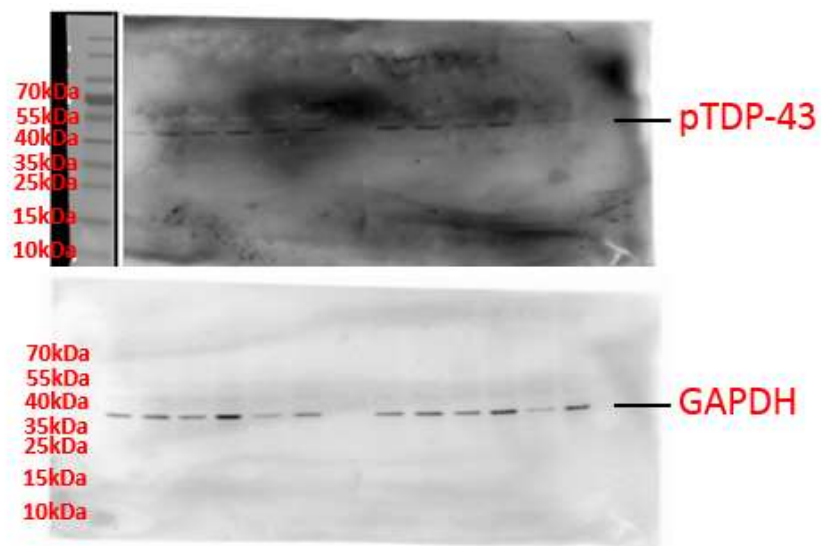

Supplement: Supplementary file 2 — Source Data for Appendix [file EMBJ-40-e106177-s002.zip › embj2020106177-sup-0002-SDataEV/embj2020106177-sup-0008-SDataFigS8.pdf]

Figure 9

Panel A

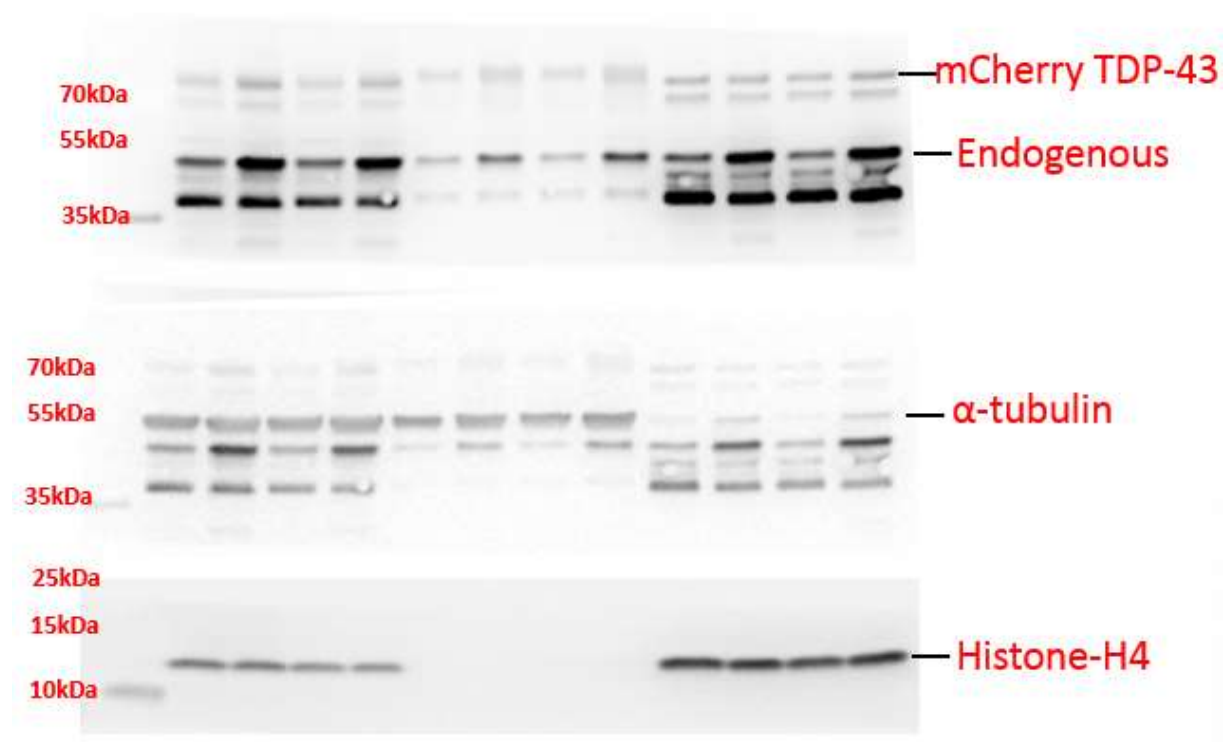

Panel F

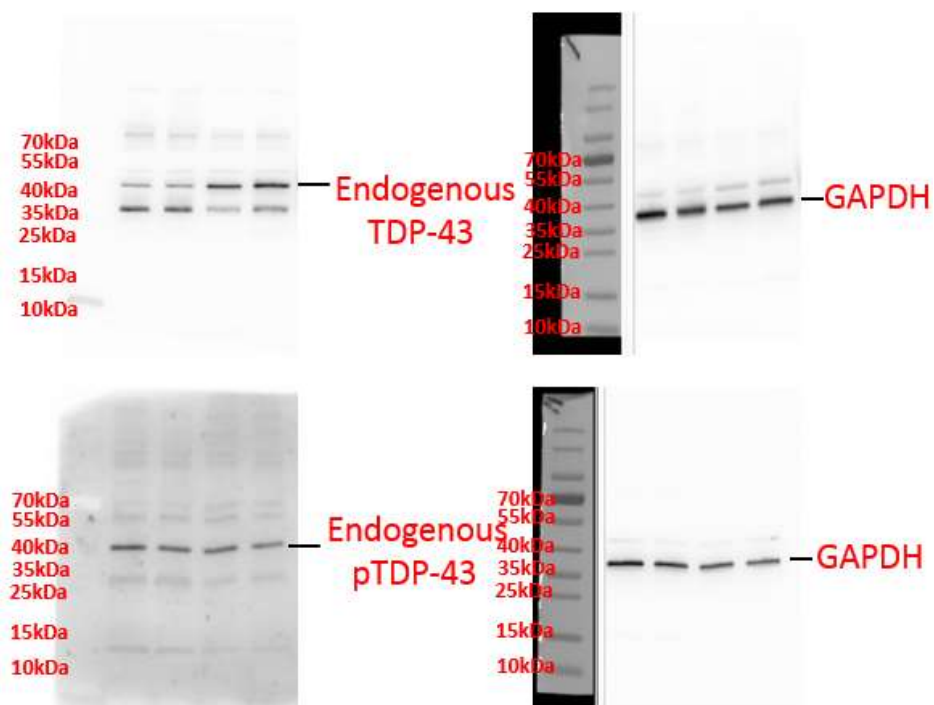

Supplement: Supplementary file 2 — Source Data for Appendix [file EMBJ-40-e106177-s002.zip › embj2020106177-sup-0002-SDataEV/embj2020106177-sup-0009-SDataFigS9.pdf]
